# Rigidity percolation uncovers a structural basis for embryonic tissue phase transitions

**DOI:** 10.1016/j.cell.2021.02.017

**Published:** 2021-04-01

**Authors:** Nicoletta I. Petridou, Bernat Corominas-Murtra, Carl-Philipp Heisenberg, Edouard Hannezo

**Affiliations:** 1 Institute of Science and Technology Austria, Klosterneuburg, Austria

**Keywords:** rigidity percolation, cell-contact network, phase transition, tissue rheology, embryo morphogenesis, cell adhesion, cell mechanics

## Abstract

Embryo morphogenesis is impacted by dynamic changes in tissue material properties, which have been proposed to occur via processes akin to phase transitions (PTs). Here, we show that rigidity percolation provides a simple and robust theoretical framework to predict material/structural PTs of embryonic tissues from local cell connectivity. By using percolation theory, combined with directly monitoring dynamic changes in tissue rheology and cell contact mechanics, we demonstrate that the zebrafish blastoderm undergoes a genuine rigidity PT, brought about by a small reduction in adhesion-dependent cell connectivity below a critical value. We quantitatively predict and experimentally verify hallmarks of PTs, including power-law exponents and associated discontinuities of macroscopic observables. Finally, we show that this uniform PT depends on blastoderm cells undergoing meta-synchronous divisions causing random and, consequently, uniform changes in cell connectivity. Collectively, our theoretical and experimental findings reveal the structural basis of material PTs in an organismal context.

## Highlights

• Zebrafish morphogenesis starts with a tissue rigidity phase transition
• Percolation theory predicts this transition based on the cell connectivity network
• Cell-cell adhesion defines the cell connectivity network and thus tissue rigidity
• Cell-cycle synchrony ensures a uniform transition by randomly changing connectivity

## Graphical abstract

**Figure:**
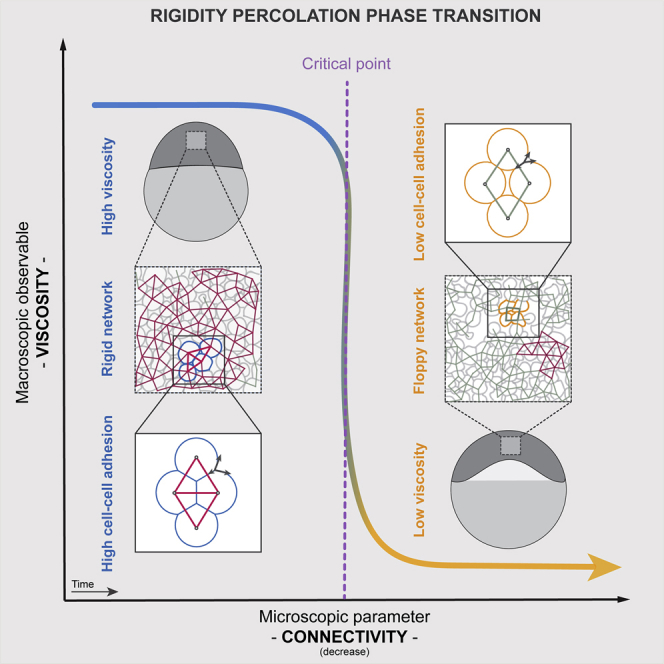

## Teaser

A general theoretical approach, rooted in cell connectivity, enables prediction of phase transitions at the tissue scale.

## Introduction

Embryonic tissues exhibit structural properties of viscoelastic materials (Forgacs et al., 1998), the precise spatiotemporal regulation of which is essential for proper embryo development (Petridou and Heisenberg, 2019). Recent direct measurements of tissue material properties, such as viscosity, yield stress, and Young’s modulus, combined with genetic and mechanical perturbations have illustrated that spatial and/or temporal changes in material characteristics can affect morphogenetic processes, such as tissue spreading and body axis elongation (Barriga et al., 2018; Mongera et al., 2018; Petridou et al., 2019). For instance, the dynamics of tissue spreading have been shown to be controlled by changes in tissue material properties without altering force application (Morita et al., 2017; Petridou et al., 2019). This suggests that regulating tissue material properties constitutes an alternative mechanism of controlling tissue morphogenesis, in addition to other more direct mechanisms, such as the regulation of force generation (Heisenberg and Bellaïche, 2013). Yet, how tissue material properties change within the developing organism remains poorly understood.

Intriguingly, changes in tissue material properties can be fast and drastic (Petridou et al., 2019), resembling phase transitions (PTs), a phenomenon playing a fundamental role in the dynamics of many complex systems (Domb and Green, 1972; Muñoz, 2017; Solé, 2011; Stanley, 1971). In physics, PTs refer to a broad class of phenomena mainly characterized by the presence of abrupt changes in some of the macroscopic properties of the system, known as “order parameters,” as a consequence of smooth variations of a “control parameter,” when the latter reaches a critical value. At such “critical points,” universal core physical features can be observed, such as singularities in macroscopic observables or power-law distributions in the statistics of order parameters (Domb and Green, 1972; Stanley, 1971). These patterns are largely independent of the specific features of the system, providing a unifying, simple mechanism determining collective behaviors of many systems of disparate nature (Muñoz, 2017; Solé, 2011).

Different classes of theoretical models have been proposed in the past decade to explore the possibility of tissues undergoing PTs. On the one hand, active particle models have been developed to explain density-dependent glassy dynamics observed during the collective migration of *in vitro* monolayers (Angelini et al., 2011; Basan et al., 2013; Garcia et al., 2015; Henkes et al., 2011; Sepúlveda et al., 2013). On the other hand, vertex models have been proposed to describe the geometrical and material properties of confluent epithelial monolayers (Alt et al., 2017; Atia et al., 2018; Bardet et al., 2013; Farhadifar et al., 2007; Fletcher et al., 2014; Kokic et al., 2019; Krajnc et al., 2018; Manning et al., 2010; Okuda et al., 2015; Siber and Ziherl, 2017). In these models, specific geometric features of cells, such as their shape arising from cell-cell adhesion and cytoskeletal forces (control parameters), trigger, when reaching a critical value, large changes in tissue rigidity (order parameter) in a density-independent manner (Bi et al., 2015; Merkel and Manning, 2018; Wang et al., 2020). Notably, in an unjammed state, cells can move at zero energy cost, thereby connecting the amplitude of relative cell movements to density-independent rigidity transitions (Bi et al., 2016). However, although such motility transitions can be observed in *in vitro* cell monolayers and some *in vivo* settings (Park et al., 2015), direct rheological measurements demonstrating actual sharp changes in tissue material properties during such (un)jamming transitions remain challenging. This has started to be addressed in 3D embryonic tissues by methods such as micropipette aspirations (MPAs) (Petridou et al., 2019) or droplets (Mongera et al., 2018), although the lack of dynamic measurements of a tissue undergoing a putative PT both in space and time impedes a proper characterization of the process. Moreover, from a statistical physics perspective, there are core universal features that are expected at critical points of tissue PTs, but they have not yet been traced in *in vivo* systems, in particular due to these experimental limitations. Finally, from a developmental biology perspective, the mechanisms underlying the robust physiological regulation of critical points *in vivo* remain largely unknown.

We have tackled these outstanding questions by analyzing fluidization of the zebrafish blastoderm, a non-confluent tissue making up the blastula (Morita et al., 2017; Petridou et al., 2019). By combining rigidity percolation theory with micropipette rheological measurements, quantitative image analysis, biophysical modeling of cell-cell contacts, and genetic perturbations, we show that the blastoderm undergoes a genuine rigidity PT, and that the cell-cell adhesion-dependent topology of the cell-cell contact network is an accurate predictor of its tissue material properties. Furthermore, we predict and observe key signatures of criticality *in vivo* and identify cell-cycle synchrony as a regulatory mechanism conferring robustness to the blastoderm when undergoing a PT.

## Results

### Cellular dynamics of zebrafish blastoderm fluidization

**Figure 1:**
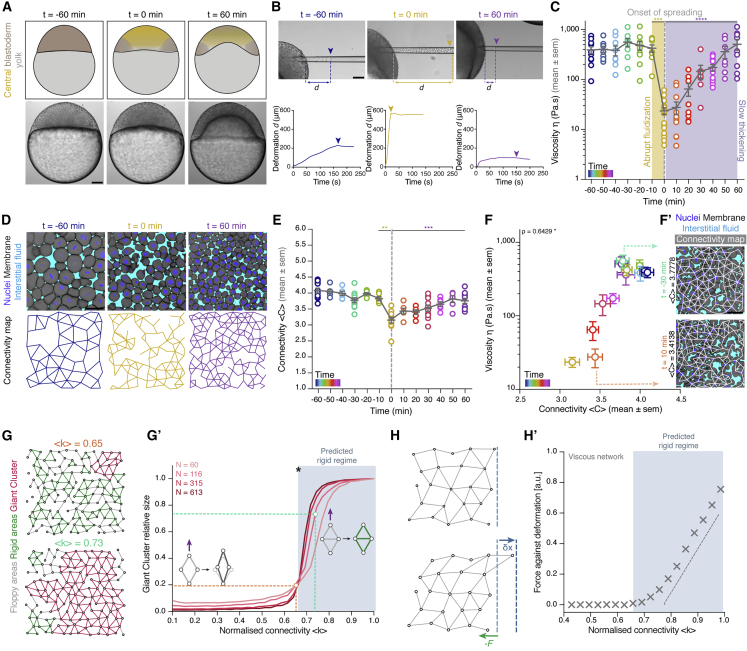
Blastoderm cell connectivity as a potential control parameter of a tissue rigidity percolation transition (A) Schematic representations (top) and bright-field single-plane images (bottom) of an exemplary embryo before (t = −60 min), at the onset (t = 0 min), and after blastoderm spreading (t = 60 min). The yellow-shaded region represents the central blastoderm. (B) Exemplary bright-field images of creep and recovery aspiration experiments in the central blastoderm at the stages described in (A) (top) and corresponding deformation (*d*) plots showing the distance covered by the tissue in the pipette under constant pressure as a function of time (bottom). Arrowheads indicate the deformation at pressure release. (C) Dot plot of individual viscosity values of the central blastoderm obtained from the aspiration experiments shown in (B) overlaid with a line plot of the mean ± SEM as a function of time (color coded for 10 min intervals) (n = 129 embryos, N = 12 embryo batches). Gray dashed line indicates the onset of blastoderm spreading during the fluidization (yellow shade)/thickening (purple shade) process. (D) Exemplary 2D confocal sections at the 1^st^–2^nd^ deep-cell layer of the blastoderm (top) and their connectivity maps (bottom) at the stages described in (A). Interstitial fluid is marked by dextran, nuclei by H2B-GFP, and membranes by membrane-red fluorescent protein (RFP). (E) Dot plot of individual connectivity <C> values (number of contacts/cell) obtained from central blastoderm confocal sections overlaid with a line plot of the mean ± SEM as a function of time (color coded) (n = 11 embryos for time points −60, −30, 0, 30, and 60 min; n = 6 embryos for all other time points; N = 11 embryo batches). Gray dashed line indicates the onset of blastoderm spreading. (F) Plot of the central blastoderm viscosity values (mean ± SEM) as a function of connectivity <C> (mean ± SEM) over time (color coded as in E; for viscosity n = 129 embryos, N = 12 embryo batches; for connectivity n = 103 blastoderms, N = 11 embryo batches). (F’) Two exemplary blastoderm confocal sections (marked as in D) with overlaid connectivity maps displaying slightly different connectivity, but by an order of magnitude different viscosity values. (G) Exemplary simulated networks with normalized connectivity <k> values above (green line in G’) and below (orange line in G’) the critical point (asterisk in G’) of the rigidity percolation transition. Floppy areas are illustrated in gray, rigid areas in green, and the giant cluster (GC) in red. (G’) Plot of the fraction of the network occupied by the GC as a function of normalized connectivity <k> in simulated random 2D triangular lattices of different sizes (N, number of nodes). The gray-shaded area indicates the network rigid regime above the critical connectivity point (k_c_, black asterisk). The schematics illustrate how, under the same deformation force (purple arrow), a floppy (left, costing zero energy) or rigid (right, costing non-zero energy due to its central bond) cluster of nodes would deform. (H) Schematic diagram of the force response (F, green arrow) for set deformation (δx, blue arrow) induced by a small displacement of the edge layer of viscous 2D networks. (H’) Plot of the force response illustrated in (H) for viscous 2D networks of size N ∼ 250 nodes, as a function of normalized connectivity <k>. Bond half-life time τ is 2Te, where Te is the number of simulation time steps. The gray-shaded area indicates the rigid regime above the k_c_, for which viscosity grows linearly as a distance from the critical point. Kruskal-Wallis test (C and E), ρ Spearman correlation test (F). Scale bars: 100 μm in (A) and (B) and 50 μm in (D) and (F’). See also Figure S1 and Video S1.

**Figure S1:**
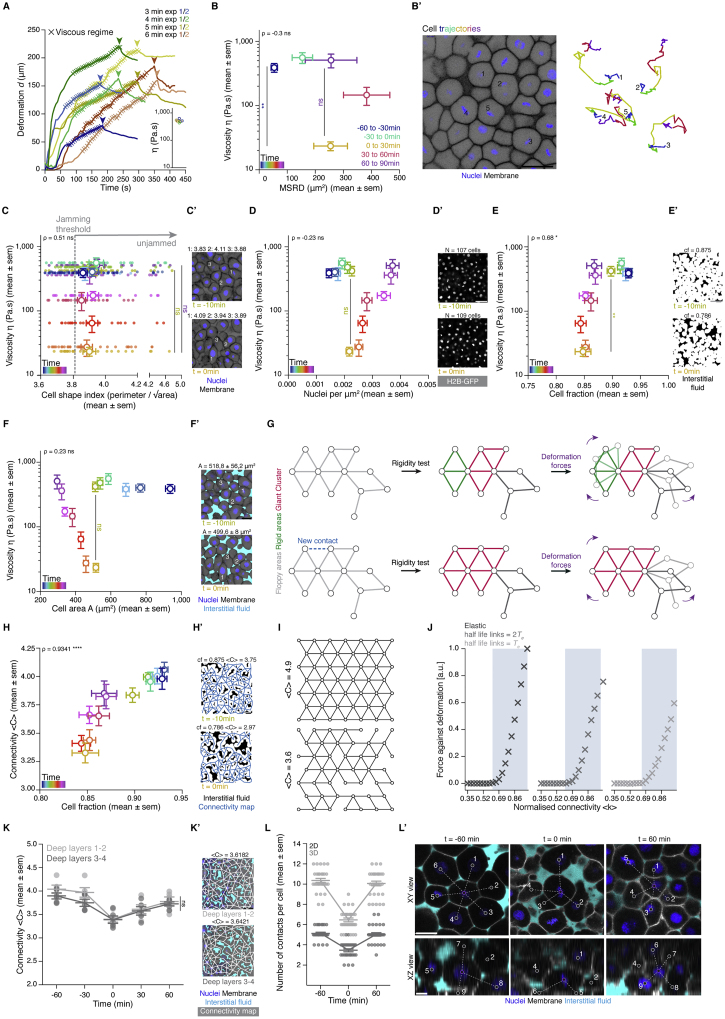
Temporal analysis of blastoderm viscosity and underlying cellular dynamics, related to Figure 1 (**A**) Plot of exemplary deformation (*d*) curves from creep and recovery aspiration experiments in the central blastoderm of sphere stage embryos (t-30min) for different aspiration times and integrated plot of the obtained viscosity values from these experiments, which found to be independent of the aspiration time. The ‘x’ signs indicate the region of the curve where the blastoderm undergoes a viscous deformation and where the slope was used for calculating the viscosity (see STAR Methods for details). Arrowheads indicate the time point of pressure release. (**B**) Plot of central blastoderm tissue viscosity (mean ± sem) as a function of the mean squared relative displacement (MSRD) of the blastoderm cells (mean ± sem), during 30min intervals from different starting time points during the fluidization/thickening process (B’). Color-code indicates 30min intervals with dark blue corresponding to viscosity at t-60min and MSRD from t-60min to t-30min, green to viscosity at t-30min and MSRD from t-30min to t0min, yellow to viscosity at t0min and MSRD from t0min to t30min, red to viscosity at t30min and MSRD from t30min to t60min and purple to viscosity at t60min and MSRD from t60min to t90min (for viscosity n = 53 embryos, N = 12 embryo batches; for MSRD n = 26 cell doublets, N = 8 embryos). Statistical tests were performed in comparison to t0min. (**B’**) Exemplary 2D confocal section at the 1^st^-2^nd^ deep cell layer of the blastoderm at t-60min (left) and color-coded 3D cell trajectories (right) for five representative cells. Nuclei are marked by H2B-GFP and membranes by membrane-RFP. (**C**) Plot of central blastoderm tissue viscosity (mean ± sem) as a function of the cell shape index (mean ± sem) (n = 390 cells, N = 3 embryos). Overlaid dot plot shows individual measurements of the cell shape index for each time point. The gray dashed line indicates the theoretical cell shape index value at which a jamming transition is predicted in epithelial tissues. Statistical tests were performed in comparison to t0min. (**C’**) Exemplary 2D confocal sections at the 1^st^-2^nd^deep cell layer of the blastoderm marked as in (B’) at t-10min and t0min, with 3 exemplary cell shape index measurements for each. (**D**) Plot of central blastoderm tissue viscosity (mean ± sem) as a function of nuclei density (mean ± sem) (n = 103 blastoderms, N = 11 embryo batches). Statistical test was performed in comparison to t0min. (**D’**) Exemplary 2D confocal sections at the 1^st^-2^nd^ deep cell layer of the blastoderm with marked nuclei by H2B-GFP at t-10min and t0min with indicated number of nuclei (N). (**E**) Plot of central blastoderm tissue viscosity (mean ± sem) as a function of cell fraction (mean ± sem) (n = 78 blastoderms, N = 6 embryo batches). Statistical test was performed in comparison to t0min. (**E’**) Exemplary binary images from the 2D confocal sections shown in (D) with marked interstitial fluid by dextran at t −10min and t 0min with indicated cell fraction (*cf.*) measurements. (**F**) Plot of central blastoderm tissue viscosity (mean ± sem) as a function of cell area (mean ± sem) (n = 652 cells, N = 6 embryos) during the fluidization/thickening process (color-coded for 10min intervals). Statistical test was performed in comparison to t0min. (**F’**) Exemplary cell area A measurements from the 2D confocal sections shown in (C) with nuclei marked by H2B-GFP, membranes by membrane-RFP and interstitial fluid by dextran, at t-10min and t0min. (**G**) Rigidity analysis of an exemplary network. Two rigid clusters (green, red) and a floppy area (gray) are identified. The shaded areas of the network depict potential response to deformation forces that would require no energy cost. Adding a single link can change the overall response of the network to deformation forces due to the sharp increase in the size of the Giant Cluster (GC) (red). (**H**) Plot of connectivity < C > (mean ± sem) as a function of cell fraction *cf.* (mean ± sem) obtained from the same 2D confocal sections of the central blastoderm during the fluidization/thickening process, color-coded for 10min intervals (n = 78 blastoderms, N = 6 embryo batches). (**H’**) Exemplary binary images from the 2D confocal sections shown in (D) with marked interstitial fluid by dextran and overlaid connectivity maps at t-10min and t0min with indicated *cf.* and < C > values. (**I**) Two exemplary triangular lattices of N = 46, L = 7, one fully connected (top panel) with < C > = 4.9, and the other one partially connected (bottom panel) with < C > 3.6, N, number of nodes, L, side length. Numerical construction of lattices starts with a fully connected lattice of certain L followed by the random removal of links until the desired average connectivity is achieved. (**J**) Plots of the linear regime of the response of the lattice against deformations as a function of normalized connectivity for an elastic lattice (left), viscous lattice with bonds half lifetime τ = 2Te (middle) and τ = Te (right), with Te being the simulation time. A permanent displacement of δx = 0.01 a.u. is applied to the top layer of nodes, while the bottom layer remains fixed - (see Figure 1H). The viscosity for viscous lattices is computed from the force exerted by the bottom layer against the deformation. The linear increase of the resistance exerted by the network starts at the critical point of the rigidity percolation (shaded area, predicted rigid regime). Parameters of the simulation are given in STAR Methods. (**K**) Dot plot of individual connectivity < C > values obtained from 2D confocal sections of the same blastoderm at the 1^st^-2^nd^ deep cell layer and at the 3^rd^-4^th^deep cell layer overlaid with a line plot of the mean ± sem as a function of time (n = 50 blastoderm networks, N = 5 embryos). (**K’**) Exemplary blastoderm confocal sections (marked as in F) at the 1^st^-2^nd^(top) and the 3^rd^-4^th^(bottom) deep cell layer with overlaid connectivity maps and indicated < C > values. (**L**) Dot plot of the number of contacts per cell as counted from XY (2D) and XYZ (3D) confocal views (mean ± sem) as a function of time (n = 30 cells, N = 3 embryos for each time point). (**L’**) Exemplary XY and XZ blastoderm confocal sections marked as in (F’). Purple circle indicates the cell chosen for counting its cell-cell contacts, gray circles indicate neighboring cells in contact to the chosen cell. Kruskal-Wallis test (B, C), Mann-Whitney test (D-F, H), ρ Spearman correlation (B-F, H). Scale bars, 50 μm (B’, D’, E’, H’, K’), 20 μm (C’, F’, L’).

In order to address whether and how embryonic tissues undergo PTs, we turned to the zebrafish blastoderm (Figure 1A). We have previously found, using MPA (Guevorkian et al., 2010; Petridou et al., 2019), that the viscosity of the central part of the blastoderm abruptly drops at the onset of morphogenesis by more than an order of magnitude within a few minutes (Figures 1A–1C, yellow box; Figure S1A; Video S1; see STAR Methods). This tissue fluidization was only transient, with the blastoderm returning to its initial viscosity values before fluidization by gradually increasing its viscosity in a slow (∼1 h) “thickening” (defined in a rheological sense as increasing viscosity) process (Figure 1C, purple box; Video S1) (Petridou et al., 2019). We first asked which microscopic cellular process may trigger such stark macroscopic blastoderm viscosity changes. Given previous reports implicating changes of cell motion and shape in density-independent unjamming transitions of confluent epithelial tissues (Bi et al., 2016; Park et al., 2015; Tetley et al., 2019), we first asked whether blastoderm fluidization might be achieved by those processes. To this end, we analyzed the mean squared relative displacement (MSRD) of blastoderm cells and cell shape index (see STAR Methods) during blastoderm tissue fluidization and thickening, but found no clearly recognizable temporal correlation between these cellular parameters and changes in blastoderm viscosity (Figures S1B, S1B’, S1C, and S1C’). This argues against the blastoderm undergoing a motion-dependent/density-independent unjamming transition. Moreover, the increase in cell number in time via proliferation, which was proposed to give rise to density-dependent jamming transitions *in vitro* (Angelini et al., 2011; Sadati et al., 2013), could not explain the viscosity changes, as analysis of blastoderm cell nuclei density did not reveal any obvious correlation with blastoderm viscosity (Figures S1D and S1D’).

Video S1. Measurements of temporal changes in central blastoderm viscosity using micropipette aspiration, related to Figure 1 Exemplary time lapse videos (5 s interval) of micropipette aspiration experiments of the central blastoderm in WT embryos before (t = −60min, t = −30min), at the onset of (t = 0 min) and after (t = 30min, t = 60 min) blastoderm spreading (left), and plots of the deformation induced by the pipette aspiration (right). Arrowheads indicate the deformation over time until the point of pressure release. Scale bar, 100 μm.

**Figure S2:**
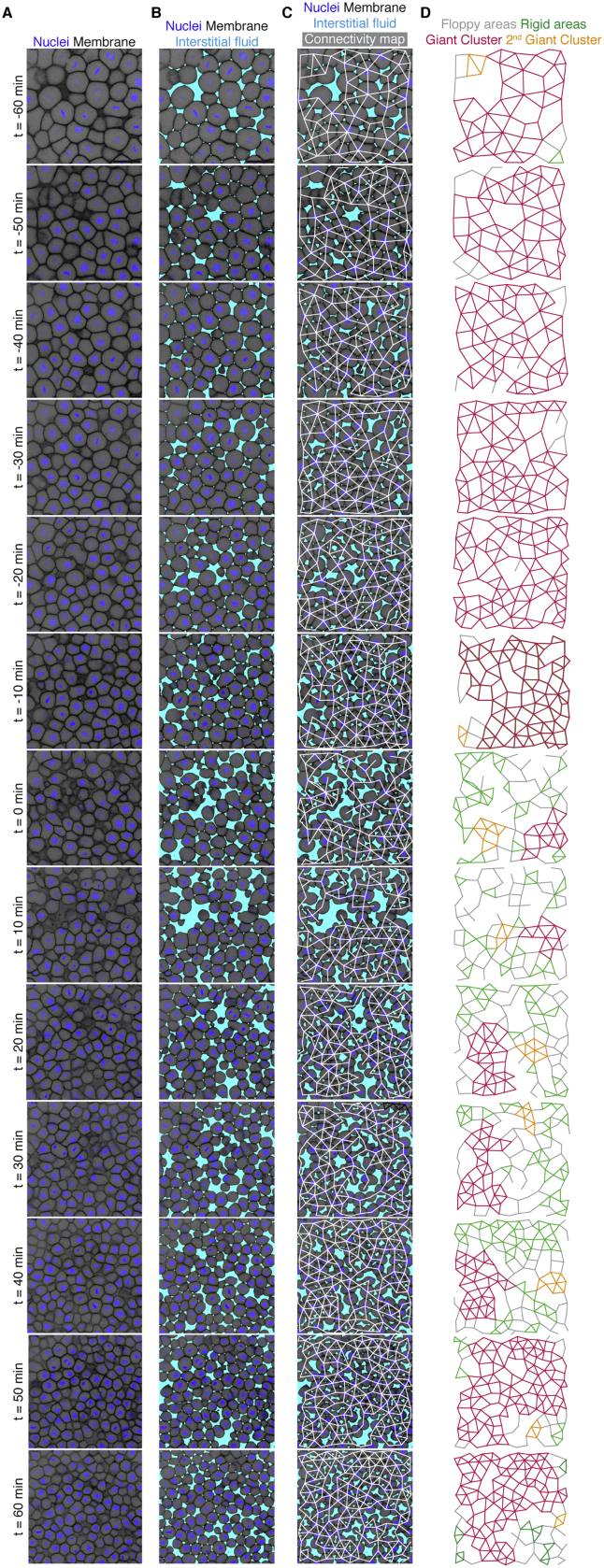
Rigidity analysis in WT embryos, related to Figure 2 (**A**) Confocal images of the central blastoderm of an exemplary WT embryo imaged at 1^st^-2^nd^deep cell layer with nuclei marked by H2B-GFP and membranes by membrane-RFP at consecutive time points during the fluidization/thickening process taken from a time lapse video of a WT embryo. (**B**) Same images as shown in (A) with labeled interstitial fluid marked by dextran to reveal the spaces between the cells. (**C**) Same images as in (B) with overlaid connectivity maps. (**D**) Rigidity analysis of the connectivity maps shown in (C). Floppy areas are illustrated in gray, rigid areas in green, the Giant Cluster (GC) in red and the 2^nd^ GC in orange.

However, we observed that, upon labeling the interstitial space within the blastoderm, cells were separated by interstitial fluid accumulations (Figures 1D, S2A, and S2B), suggesting that the blastoderm tissue is non-confluent. Importantly, when expressing cell density not as nuclear density, but as the percentage of space not occupied by interstitial fluid (cell fraction), we detected a clear nonlinear relationship between cell fraction and tissue viscosity, independent of variations in cell size (Figures S1E, S1E’, S1F, and S1F’). Such a nonlinear relationship between cell fraction and tissue viscosity is reminiscent of the physics of granular sphere packings, where increasing the packing fraction above a critical point triggers a jamming transition, characterized by the appearance of a non-zero shear modulus despite structural disorder. This occurs when contacting spheres are close enough to form a “rigid” graph of contacts, so that displacing any such spheres requires energy expenditure (see schematic in Figure S1G) (van Hecke, 2010; Jacobs and Thorpe, 1996). Thus, we sought to explore this analogy in a biological setting by creating a temporal map of cellular connectivity, with cell-cell contacts being defined by the absence of interstitial fluid between neighboring cells (Figures 1D, 1E, and S2C). We found that blastoderm cell connectivity was directly related to cell fraction (Figures S1H and S1H’) and consequently displayed a nonlinear relationship with blastoderm viscosity (Figure 1F). Specifically, before blastoderm fluidization, when blastoderm tissue viscosity was largely unchanged, cell connectivity was smoothly and gradually reduced (Figures 1E, 1F, and 1F’, blue-green time points). This reduction in cell connectivity continued up to a threshold value of ∼3–4 contacts per cell, when blastoderm viscosity abruptly dropped at the onset of morphogenesis (Figures 1E, 1F, and 1F’, yellow time points), followed by a slow increase in cell connectivity during blastoderm thickening (Figures 1E, 1F, and 1F’, purple time points). Intriguingly, this transition point of connectivity is very close to the one predicted by rigidity percolation theory (two-thirds of maximal average number of contacts) separating floppy versus rigid connectivity graphs (Jacobs and Thorpe, 1995, 1996; Laman, 1970; Maxwell, 1864; Thorpe, 1983), suggesting that the blastoderm might undergo a rigidity percolation transition.

### Rigidity percolation as a predictor for *in vivo* tissue material properties

To test whether the blastoderm indeed undergoes a rigidity percolation transition, we first generated *in silico* 2D triangular graphs of different sizes and connectivity levels typically used to characterize the network of contacts in a standard sphere packing (Figure S1I) and performed a rigidity analysis to determine which tissue regions are expected to be rigid (Figure 1G). A cluster is considered as rigid when all its degrees of freedom are absorbed by the structure and thus cannot be deformed without expense of energy (Figure 1G’, green cluster). As previously described (Jacobs and Thorpe, 1995, 1996), we found above the critical point of two-thirds of the maximum average connectivity the appearance of a rigid “giant cluster” (GC), which percolated over the size of almost the entire system (Figures 1G, 1G’, and S1G, red cluster). In this framework, the average connectivity of the network represents the control parameter, with two-thirds of the maximum connectivity (i.e., ∼4 contacts per cell), constituting the critical point for a rigidity PT. The size of the GC, in turn, corresponds to the order parameter of the system, being small below the critical point and close to the size of the system above it (Figures 1G and G’). Crucially, the presence of a GC implies that the network will resist any deformation, whereas in the absence of the GC, deformations and rearrangements of network nodes and regions are possible at no energy cost (Figure S1G). Therefore, cell connectivity, and thus the size of the GC, might represent a simple putative criterion for predicting the material property of a tissue, and corresponding PT, from purely topological metrics.

Notably, classical rigidity percolation considers networks of elastic springs, giving rise to zero elastic modulus below the critical point and linearly increasing shear modulus as a function of distance above it (van Hecke, 2010). By contrast, the abrupt tissue material changes that we observed within the blastoderm are changes in viscosity (Petridou et al., 2019). Such viscous response of tissues can arise from turnover or re-arrangements (Cavanaugh et al., 2020; David et al., 2014; Iyer et al., 2019; Ranft et al., 2010). To incorporate this behavior in a minimal way within our rigidity percolation network analysis, we analyzed 2D networks of elastic springs where the bonds can spontaneously break and heal (with a characteristic timescale τ; see STAR Methods for details of this modeling strategy), revealing that the critical point of rigidity percolation triggers a transition from low to high viscosity (Figures 1H, H’, and S1J; see STAR Methods). This suggests that rigidity percolation can be used to describe the changes in tissue viscosity observed within the blastoderm.

**Figure 2:**
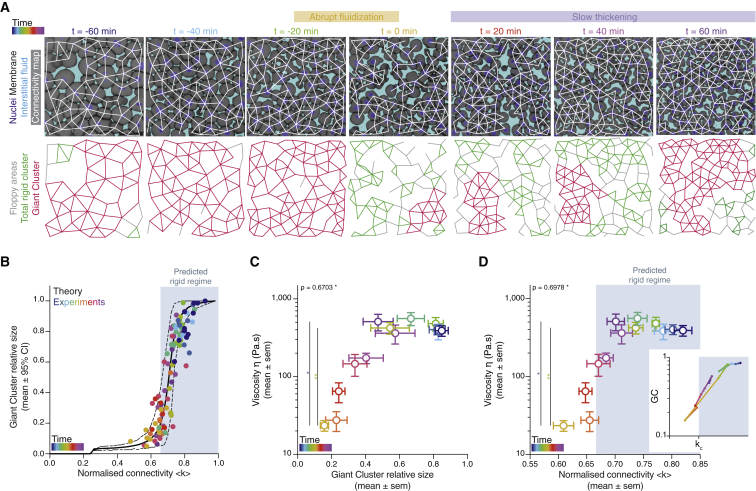
A tissue rigidity percolation transition describes the temporal blastoderm viscosity profile (A) Exemplary 2D confocal sections at the 1^st^–2^nd^ deep-cell layer of the central blastoderm with overlaid connectivity maps (top) and their rigidity profile (bottom) at consecutive time points during the fluidization/thickening process (color coded). Interstitial fluid is marked by dextran, nuclei by H2B-GFP, and membranes by membrane-RFP. Floppy areas are illustrated in gray, rigid areas in green, and the rigid GC in red. Shaded yellow and purple areas indicate the time period of tissue fluidization and thickening, respectively. (B) Plot of the fraction of the network occupied by the GC (mean ± 95% confidence interval [CI]) as a function of normalized connectivity <k> in simulated random networks of the same size as the average size of experimental networks (black). Overlaid dot plot of the measured GC sizes as a function of the normalized connectivity <k> for experimental networks of the central blastoderm at different time points during the fluidization/thickening process (color coded for 10 min intervals) (n = 103 experimental networks, N = 11 embryo batches), agreeing with the theoretical expectation. (C) Plot of tissue viscosity (mean ± SEM) as a function of the GC relative size (mean ± SEM) for experimental networks of the central blastoderm at different time points during the fluidization/thickening process (color coded as in B) (for viscosity n = 129 embryos, N = 12 embryo batches; for GC n = 103 blastoderms, N = 11 embryo batches). Statistical tests were performed in comparison to t = 0 min. (D) Plot of tissue viscosity (mean ± SEM) as a function of normalized connectivity <k> (mean ± SEM) for the samples described in (C) (for viscosity n = 129 embryos, N = 12 embryo batches; for normalized connectivity <k> n = 103 blastoderms, N = 11 embryo batches). Statistical tests were performed in comparison to t = 0 min. The integrated plot illustrates the time trajectory (color coded) of the central blastoderm material phase state (relative size of GC) as a function of its connectivity (k_c_). The gray-shaded region in (B) and (D) indicates the rigid regime above the k_c_. Kruskal-Wallis test (C and D), ρ Spearman correlation test (C and D). Scale bars: 50 μm in (A). See also Figure S2, Table S1, and Video S2.

To directly test the relationship between cell connectivity, GC size, and tissue viscosity *in vivo*, we sought to compare simulated random networks with experimental 2D cell connectivity networks within the blastoderm. We reasoned that a 2D analysis is applicable for the 3D tissue context of the blastoderm, given that no systematic changes in 2D network connectivity were found between 2D sections of the blastoderm at different heights within the tissue (Figures S1K and S1K’) and that measuring average connectivity changes in 3D showed a very similar pattern as observed in 2D (Figures S1L and S1L’), with a decrease of connectivity at fluidization up to a value close to that expected for the critical point of a rigidity PT in 3D networks (Chubynsky and Thorpe, 2007). We then compared simulated random 2D networks of similar average size with those typically imaged within the blastoderm (∼100 cells per network) (Figures 2A and 2B; see STAR Methods). We found that not only the relationship between GC size and network connectivity was conserved between simulated and experimentally observed connectivity networks (Figures 2A and 2B), but also that the temporal change in the experimentally observed size of the GC in such networks (Figures 2A and S2D; Video S2) closely matched the temporal changes in tissue viscosity measured during the blastoderm fluidization/thickening process (Figure 2C): high tissue viscosity (before fluidization and after thickening) corresponded to rigid networks with connectivity above the critical point and large GCs, and low tissue viscosity (during fluidization) corresponded to floppy networks with connectivity below the critical point and small GCs (Figures 2C and 2D). Taken together, these findings show that the changes in blastoderm viscosity can be explained by changes in cell connectivity, which control overall tissue rigidity, as predicted by rigidity percolation theory.

Video S2. Analysis of temporal changes of central blastoderm rigidity in WT embryos using rigidity percolation, related to Figures 2 and 5 Exemplary time lapse video (3 min interval) of the central blastoderm of a WT embryo with overlaid connectivity maps (left) and rigidity analysis (right). Nuclei are marked by H2B-GFP, membranes by membrane-RFP and interstitial fluid by dextran. Floppy areas are illustrated in gray, rigid areas in green, the Giant Cluster (GC) in red and the 2^nd^ GC in orange. Rigid time points are marked in purple, floppy time points in yellow. Scale bar, 50 μm.

### Perturbation experiments reveal conserved dependency of tissue rheology on cell connectivity

**Figure S3:**
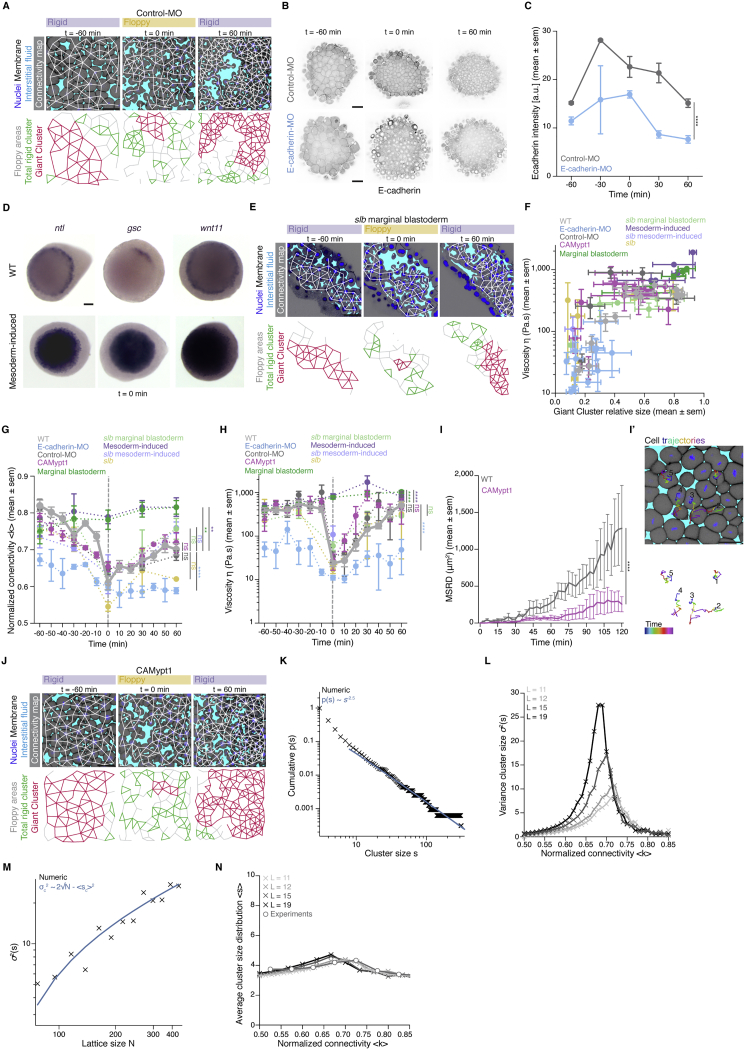
Experimental manipulations of connectivity, topological rigidity, and tissue viscosity, related to Figure 3 (**A**) Exemplary 2D confocal sections at the 1^st^-2^nd^deep-cell layer of the central blastoderm of a control-morpholino (MO) injected embryo with overlaid connectivity maps (top) and their rigidity profile (bottom) at consecutive time points during the fluidization/thickening process. Interstitial fluid is marked by dextran, nuclei by H2B-GFP and membranes by membrane-RFP. Floppy areas are illustrated in gray, rigid areas in green, the Giant Cluster (GC) in red. (**B**) Exemplary 2D confocal sections of the central blastoderm of a control-MO and an *e-cadherin*-MO injected embryo immunostained for E-cadherin. (**C**) Plot of E-cadherin protein levels (mean ± sem) as judged by the fluorescence intensity of the immunostaining experiments as a function of time (control-MO, n = 18 embryos; *e-cadherin*-MO, n = 16 embryos, N = 2 embryo batches). (**D**) Exemplary top views of *ntl*, *gsc* and *wnt11* expression in zebrafish WT and mesoderm-induced embryos at the onset of blastoderm spreading (t0min). (**E**) Exemplary 2D confocal sections at the 1^st^-2^nd^deep-cell layer of the marginal blastoderm of a *slb/wnt 11*mutant embryo marked as in (A) with overlaid connectivity maps (top) and their rigidity profile labeled as in (A) (bottom) at consecutive time points during the fluidization/thickening process. (**F**) Plot of blastoderm tissue viscosity (mean ± sem) as a function of the fraction of the network occupied by the GC (mean ± sem) for the experimental conditions described in Figure 3D (for viscosity: central blastoderm of WT n = 129, N = 11; *e-cadherin*-MO n = 94, N = 6; control-MO n = 71, N = 6; CAMypt1 n = 66, N = 7; *slb/wnt11 f2* mutant n = 54, N = 4; mesoderm-induced WT n = 42, N = 6; mesoderm-induced *slb/wnt11 f2* mutant n = 13, N = 3 embryos; marginal blastoderms of WT n = 115, N = 9; *slb/wnt11 f2* mutant n = 44, N = 5 embryos; for GC: sample number as described in Figure 3D; n, number of embryos, N, number of embryo batches). (**G**) Plot for normalized connectivity < k > (mean ± sem) as a function of time for central blastoderm of WT (n = 103, N = 11), *e-cadherin*-MO (n = 54, N = 6), control-MO (n = 15, N = 3), CAMypt1 (n = 89, N = 13), *slb/wnt11 f2* mutant (n = 10, N = 2), mesoderm-induced WT (n = 15, N = 3) and for the marginal blastoderm of WT (n = 15, N = 3) and *slb/wnt11 f2* mutant (n = 15, N = 3) embryos. n, number of networks, N, number of embryos. Grey dashed line indicates the onset of blastoderm spreading. (**H**) Plot of tissue viscosity values (mean ± sem) as a function of time for central blastoderm of WT (n = 129, N = 11), *e-cadherin*-MO (n = 94, N = 6), control-MO (n = 71, N = 6), CAmypt1 (n = 66, N = 7), *slb/wnt11 f2* mutant (n = 54, N = 4), mesoderm-induced WT (n = 42, N = 6), mesoderm-induced *slb/wnt11 f2* mutant (n = 13, N = 3) and for marginal blastoderm of WT (n = 115, N = 9), *slb/wnt11 f2* mutant (n = 44, N = 5) embryos. n, number of embryos, N, number of embryo batches. Grey dashed line indicates the onset of blastoderm spreading. (**I**) Plot of the MSRD (mean ± sem) for blastoderm cells of CAMypt1 overexpressing embryos as a function of time (with −60min as reference time point) during the fluidization/thickening process and (**I’**) an exemplary 2D confocal section at the 1^st^-2^nd^deep cell layer of a CAMypt1 overexpressing blastoderm marked as in (A) at t-60min with color-coded (for 30min intervals) 3D cell trajectories (n = 10 cell doublets each, N = 3 embryos each). (**J**) Exemplary 2D confocal sections at the 1^st^-2^nd^deep-cell layer of the central blastoderm of an embryo overexpressing CAMypt1 marked as in (A) with overlaid connectivity maps (top) and their rigidity profile labeled as in (A) (bottom) at consecutive time points during the fluidization/thickening process. (**K**) Power-law distribution of cluster sizes near the critical point, p(s). The cluster size around the peak of the variance of this distribution, located at around k = 0.68, was computed from an ensemble of lattices with L = 35, N = 1208 (L, side length; N, number of nodes). The blue line shows the slope of a power law with exponent ∼-2.5. (**L**) Evolution of variance in rigid cluster size $\sigma^{2}\left(s\right)$ (for clusters other than the GC) along the connectivity values for different lattice sizes (L = 11; 12; 15; 19, N = 116; 139; 218; 352). A clear peak is observed close to the critical point, whose strength grows in size, with the peak displaying a small drift toward higher values than 2/3 of < k > for very small systems due to the increasing role of the boundaries containing nodes with less incident links. (**M**) Evolution of $\sigma^{2}\left(s\right)$ as a function of the lattice size N, showing a well-defined dependence $\sigma^{2}\left(s\right)\propto \sqrt{N}$. The prediction given in Equation 1 of the STAR Methods, is plotted in blue. (**N**) Evolution of the average rigid cluster size < s > (for clusters other than the GC) along the connectivity values for the same lattice sizes as in (L). Contrary to what is observed for $\sigma^{2}\left(s\right)$, < s > has a stable behavior across different connectivity values and displays a convergent behavior as a function of different network sizes. The average rigid cluster size as a function of their normalized connectivity for experimental networks (described in Figure 3F) is plotted with gray circles, showing good agreement with the simulated networks and lacking convergence. Kruskal-Wallis test (G, H), Mann-Whitney test (C, I). Scale bars, 50 μm (A, B, E, I’, J), 100 μm (D).

**Figure 3:**
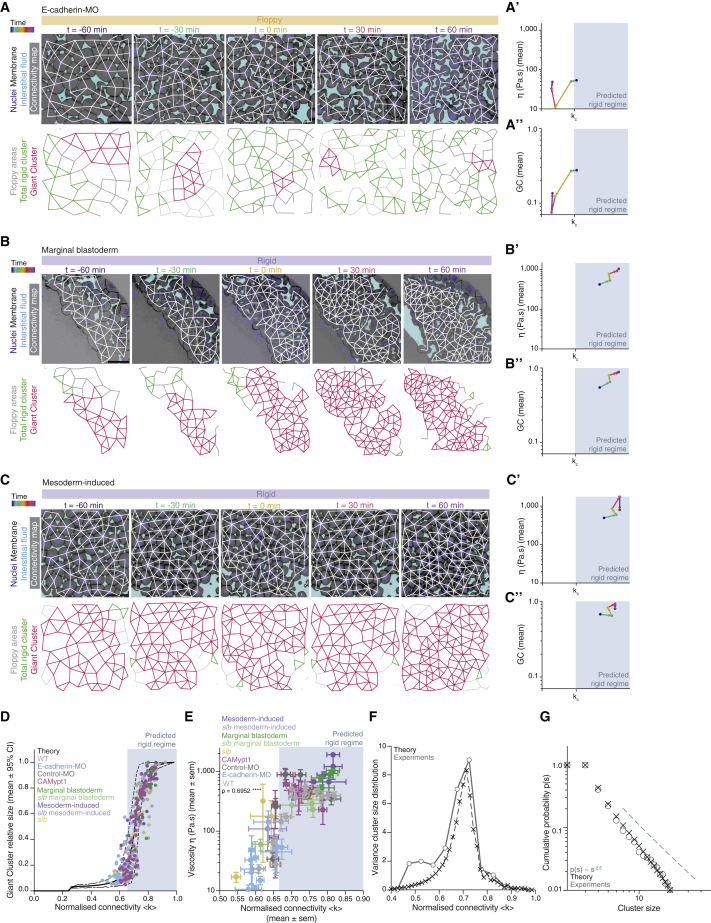
The blastoderm connectivity profile identifies key hallmarks of criticality during its rigidity percolation PT (A–C) Exemplary 2D confocal sections at the 1^st^–2^nd^ deep-cell layer of the central blastoderm of an *e-cadherin*-*morpholino* (MO)-injected embryo (A), of the marginal blastoderm of a WT embryo (B), and of the central blastoderm of a mesoderm-induced embryo (C) with overlaid connectivity maps (top) and their rigidity characteristics (bottom) during the fluidization/thickening process (color coded for 30 min intervals). Interstitial fluid is marked by dextran, nuclei by H2B-GFP, and membranes by membrane-RFP. Floppy areas are illustrated in gray, rigid areas in green, and the GC in red. Yellow- and purple-shaded areas indicate fluidized and rigid blastoderms, respectively. (A’, B’, and C’) Plots of the time trajectory (color coded) of blastoderm viscosity (mean) as a function of its normalized connectivity <k> for *e-cadherin*-MO central blastoderm (A’; for viscosity n = 94, N = 6; for connectivity n = 54, N = 6), WT marginal blastoderm (B’; for viscosity n = 115, N = 9; for connectivity n = 15, N = 3), and mesoderm-induced central blastoderm (C’; for viscosity n = 42, N = 6; for connectivity n = 15, N = 3). (A’’, B’’, and C’’) Plots of the time trajectory (color coded) of the GC relative size as a function of its normalized connectivity <k> for the samples described in (A’), (B’), and (C’). (D) Plot of the fraction of the network occupied by the GC (mean ± 95% CI) as a function of normalized connectivity <k> in simulated random networks of the same size as the average size of WT experimental networks (black). Overlaid dot plot of the measured GC size as a function of the normalized network connectivity <k> of the central blastoderm in WT (n = 103, N = 11), *e-cadherin*-MO (n = 54, N = 6), control-MO (n = 15, N = 3), CAMypt1 (n = 89, N = 13), *slb/wnt11f2* mutant (n = 10, N = 2), and mesoderm-induced WT (n = 15, N = 3) embryos and of the marginal blastoderm in WT (n = 15, N = 3) and *slb/wnt11f2* mutant (n = 15, N = 3) embryos. n, number of networks; N, number of embryos. (E) Plot of central blastoderm tissue viscosity (mean ± SEM) as a function of normalized connectivity <k> (mean ± SEM) for the experimental networks described in (D) (for viscosity: central blastoderm of WT n = 129, N = 11; *e-cadherin*-MO n = 94, N = 6; control-MO n = 71, N = 6; CAMypt1 n = 66, N = 7; *slb/wnt11f2* mutant n = 54, N = 4; mesoderm-induced WT n = 42, N = 6; mesoderm-induced *slb/wnt11f2* mutant n = 13, N = 3 embryos; marginal blastoderms of WT n = 115, N = 9; *slb/wnt11f2* mutant n = 44, N = 5 embryos; for connectivity: samples described in D). (F) Plot of the variance (Var) of the distribution of rigid cluster sizes p(s) other than the GC, as a function of their normalized connectivity <k>, in simulated networks of the same size as the average size of experimental networks (black) and in the experimental networks described in (D) (gray) (except marginal networks), showing divergence at the critical point, with good theory-experiment agreement. (G) Plot of the cumulative distribution of rigid cluster sizes p(s) other than the GC near the critical point. The numerical experiment shows the scaling behavior of cluster size distribution p(s) for networks of arbitrary large size (∼1,200 nodes). The overlaid plot shows the cluster size distribution near criticality for real networks, showing excellent agreement with predictions. The dashed line shows a power-law p(s) ∼ s^−2.5^. The gray-shaded regions at the plots indicate the rigid regime above the theoretical k_c_. ρ Spearman correlation test (E). Scale bars: 50 μm in (A)–(C). See also Figure S3 and Table S1.

We next sought to experimentally perturb blastoderm cell connectivity to challenge the percolation model and address how reliably cell connectivity can predict tissue rheology (Table S1). To modulate cell connectivity, we interfered with cell-cell adhesion directly by reducing the levels of E-cadherin expression via injection of *e-cadherin-morpholino* (MO) (Figures S3B and S3C) (Babb and Marrs, 2004) and indirectly by analyzing different regions of the blastoderm or changing cell fate specification within the blastoderm. Directly interfering with cell connectivity by diminishing E-cadherin expression reduced global cell network connectivity within the blastoderm (Figures 3A and S3G) and, as predicted by the theory, GC size to values below the critical point during the period when control-MO-injected embryos were undergoing tissue fluidization/thickening (Figures 3A, 3A’’, 3D, and S3A–S3C). Importantly, measurement of tissue viscosity in *e-cadherin* morphant embryos during this period also revealed low values of viscosity, as expected for networks below the critical point within the floppy regime (Figures 3A’, 3E, S3F, and S3H).

Furthermore, we had previously shown that the blastoderm margin, in contrast to the blastoderm center, does not undergo tissue fluidization and that this is due to the restricted expression of Wnt/PCP pathway components within the margin (Figure S3D) (Petridou et al., 2019). Consistent with our previous observations, we found that cell connectivity remained persistently high in the blastoderm margin during the period when the blastoderm center was undergoing fluidization (Figures 3B and S3G). As theoretically expected, we found that this high level of connectivity was accompanied by the presence of large GCs and high tissue viscosity (Figures 3B–3B’’, 3E, S3F, and S3H). By contrast, marginal tissues of mutant embryos for the Wnt/PCP pathway component *wnt11f2* (*slb*) (Heisenberg et al., 2000), which undergo marginal tissue fluidization in a similar manner as the blastoderm center (Figures S3E, S3F, and S3H), displayed low connectivity values (Figure S3G) and small GCs (Figures 3E, S3E, and S3F).

Finally, to indirectly interfere with cell connectivity within the blastoderm by changing cell fate specification, we ectopically induced mesodermal cell fate, which in wild-type (WT) embryos is confined to the blastoderm margin, in the blastoderm center (Figure S3D). Given that mesoderm specification induces the expression of Wnt/PCP components within the blastoderm margin (Figure S3D) (Makita et al., 1998) and, consequently, high cell connectivity and tissue viscosity within this area, we predicted and found that ectopic mesoderm induction in the blastoderm center not only abrogated tissue fluidization but also raised the level of cell connectivity consistently above the critical point during the period when control embryos underwent tissue fluidization and thickening (Figures 3C–3C’’, 3E and S3F–S3H). Collectively, our perturbation experiments show that all of the tested blastoderm cell connectivity networks collapse on the same master curves describing the relationship between cell connectivity, GC size (Figure 3D), and tissue rheology (Figure 3E), suggesting that rigidity percolation analysis reliably predicted changes in tissue viscosity on the basis of associated changes in cell connectivity within the blastoderm. This central role of cell connectivity in determining tissue viscosity was further confirmed by our observation that interfering with blastoderm cell motility (Figures S3I and S3I’), but not connectivity (Figure S3J), by mildly reducing myosin II activity in those cells, had no recognizable effect on GC size and blastoderm viscosity (Figures 3E and S3F–S3H).

### Hallmarks of a rigidity percolation PT *in vivo*

A defining feature of high-order PTs is the divergence of specific observables near critical points as well as the appearance of power-law distributions in observables related to the order parameter (Domb and Green, 1972; Solé, 2011; Stanley, 1971). Our simulations showed a clearly defined power-law pattern in the distribution of cluster sizes other than the GC close to the critical point, with an exponent around 2.5 (Figures 3G and S3K; see STAR Methods for fitting techniques). This critical exponent implies that the variance of rigid cluster sizes (outside the GC) should diverge at the critical point in infinite systems. In the case of finite systems, traces of this singularity remained as a sharp peak in the variance of rigid cluster sizes around the critical point, whose height, given the critical exponent found, should diverge with system size as ∼N^1/2^, where N is the number of nodes (Figures S3L and S3M; see STAR Methods). The average of rigid cluster sizes, on the contrary, is nearly constant across system sizes and connectivity values (Figure S3N; see STAR Methods). To test the existence of such universal features within the blastoderm, we compared the average and variance of rigid cluster sizes as a function of connectivity between experimental and theoretical networks, which revealed a close match in a parameter-free manner (Figures 3F and S3N). We then went one step further and computed the experimental rigid cluster size distribution outside the GC for 30 networks from all of the above-described experimental conditions lying near the critical point (see STAR Methods). As predicted by the numerical experiments, and despite the relatively small size of the system, the experimental cluster size distribution near criticality closely followed the theory, displaying a power law with exponent around −2.5 with a correction for small cluster sizes (Figure S3K; note that this exponent is well defined only for large systems; see STAR Methods for details) (Hanel et al., 2017) (Figure 3G; STAR Methods). Collectively, these findings reveal key hallmarks of criticality in the blastoderm, further supporting the hypothesis that the blastoderm indeed undergoes a rigidity percolation PT.

### Cell-cell adhesion defines cell connectivity and blastoderm rigidity

**Figure 4:**
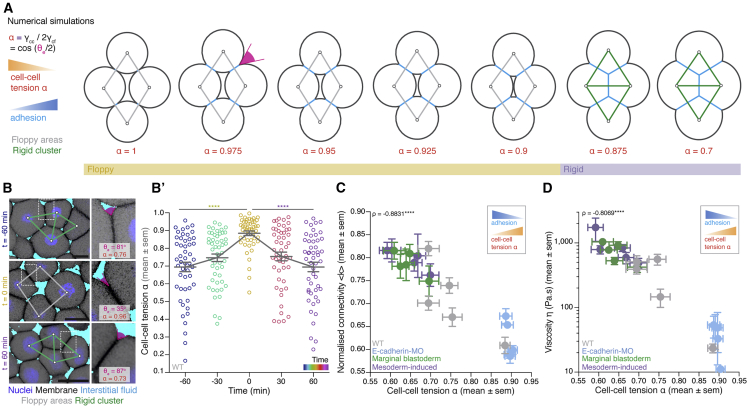
Cell-cell adhesion defines cell connectivity and blastoderm rigidity (A) Numerical simulations of a mechanical toy model for the morphology of a 4-cell rhombus cluster. Increasing cell-cell adhesion (blue) by decreasing cell-cell tension α (orange) results in contact size expansion and increased connectivity, promoting the emergence of rigid connectivity motifs (green cluster). Contact angle $\theta_{e}$ (magenta) is used to infer α. Floppy areas are illustrated in gray and rigid areas in green. Yellow- and purple-shaded areas indicate floppy and rigid clusters, respectively (exact values of the α threshold slightly depend on the initial configuration of cells). (B) Exemplary high-magnification 2D confocal sections at the 1^st^–2^nd^ deep-cell layer of the central blastoderm at consecutive time points overlaid with their rigidity profile during the fluidization/thickening process, with close-ups of exemplary contact angle $\theta_{e}$ measurements (right, magenta) and calculated cell-cell tension α. Interstitial fluid is marked by dextran, nuclei by H2B-GFP, and membranes by membrane-RFP. Floppy areas are illustrated in gray and rigid areas in green. (B’) Dot plot of individual cell-cell tension α measurements in the central blastoderm obtained from the experiments shown in (B) overlaid with a line plot of the mean ± SEM as a function of time (color coded) (n = 50 cell-cell contacts, N = 4 embryos per time point). (C) Plot of normalized connectivity <k> (mean ± SEM) as a function of cell-cell tension α (mean ± SEM) for several experimental conditions during the fluidization/thickening process (for connectivity: central blastoderm of WT n = 55, N = 11; *e-cadherin*-MO n = 30, N = 6; mesoderm-induced n = 15, N = 3; marginal blastoderm n = 15, N = 3; n, number of blastoderms, N, number of embryos; for cell-cell tension α: n = 50 cell contacts, N = 4 embryos each data point). (D) Plot of viscosity (mean ± SEM) as a function of cell-cell tension α (mean ± SEM) for the experimental conditions described in (C) during the fluidization/thickening process (for cell-cell tension α: n = 50 cell contacts, N = 4 embryos each data point; for viscosity: central blastoderm of WT n = 53, N = 11; *e-cadherin*-MO n = 54, N = 6; mesoderm-induced n = 42, N = 6; marginal blastoderms of WT n = 115, N = 9; n, number of embryos; N, number of embryo batches). Kruskal-Wallis test (B’), ρ Spearman correlation test (C and D). Scale bars: 20 μm in (B). See also Figure S4.

Our results so far demonstrate that average cell-cell contact number is sufficient to predict global contact topology (such as GC size), which itself serves as a reliable predictor for tissue material properties across time and experimental perturbations. But how is contact topology regulated from a biomechanical and biophysical perspective? Our previous findings had shown that cell-cell adhesion decreased due to mitotic rounding at the onset of blastoderm fluidization (Petridou et al., 2019). To evaluate the role of cell-cell adhesion in determining global contact topology, we developed a mechanical toy model of a 2D cluster of 4 cells organized in a rhombus, one of the simplest non-rigid topologies (Figures 1G’ and 4A), based on previous studies of cell-cell adhesion and embryo compaction (David et al., 2014; Maître et al., 2012, 2015). We considered constant cell volume, so that the ratio $\alpha =\gamma_{cc}/2\gamma_{cf}$ of cell-cell tension ($\gamma_{cc}$) to cell-fluid tension ($\gamma_{cf}$), which defines cell-cell adhesion strength, is the single parameter specifying cluster configuration (Figure 4A). For α>1, cells are non-adhesive as they round up to minimize their total surface energy, while for α<1, cells become adhesive by forming cell-cell contacts that result in surface energy minimization. Consequently, increasing cell-cell adhesion (decreasing α) in our 2D cluster of 4 cells organized in an initially floppy rhombus (Figure 4A, gray cluster, floppy regime) led to cell-cell contact expansion up to the point where tricellular junctions appeared, forming an additional contact in the cluster that collapsed the central interstitial space and transformed the configuration into a rigid topological structure (Figure 4A, green cluster, rigid regime).

**Figure S4:**
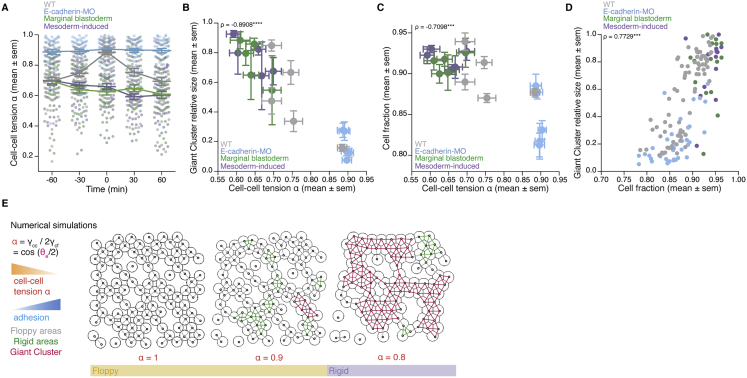
Relationship between cell-cell adhesion, rigidity percolation, and cell fraction, related to Figure 4 (**A**) Dot plot of individual cell-cell tension α measurements of the experimental conditions described in Figure 4C at consecutive time points during the fluidization/thickening process (n = 50 cell-cell contact angles per experimental condition and time point). (**B**) Plot of the fraction of the network occupied by the GC (mean ± sem) as a function of cell-cell tension α (mean ± sem) for the experimental conditions described in Figure 4C at different time points during the fluidization/thickening process (for cell-cell tension α: n = 50, N = 4, n, number of cell-cell contact angles, N, number of embryos per experimental condition and time point; for GC: WT, n = 55, N = 11; E-cadherin-MO, n = 30, N = 6; Marginal blastoderm, n = 15, N = 3; Mesoderm-induced, n = 15, N = 3, n, number of blastoderms, N = number of embryos). (**C**) Plot of the blastoderm cell fraction (mean ± sem) as a function of cell-cell tension α (mean ± sem) for the experimental conditions described in Figure 4C at different time points during the fluidization/thickening process (for cell-cell tension α: n = 50 cell-cell contact angles per experimental condition and time point; for cell fraction: n = 4 blastoderms per experimental condition and time point). (**D**) Plot of the fraction of the network occupied by the GC as a function of the cell fraction for the experimental conditions described in Figure 4C at different time points during the fluidization/thickening process (WT n = 78, N = 11; *e-cadherin*-MO n = 39, N = 3; blastoderm margin n = 15, N = 3; mesoderm-induced n = 15, N = 3; n, number of blastoderms; N, number of embryo batches). (**E**) Numerical simulations and overlaid connectivity and rigidity maps of randomly packed soft spheres of comparable size to the experimental data (N = 100) at different cell-cell tension α values. Floppy areas are illustrated in gray, rigid areas in green and the GC in red. Yellow and purple shaded areas indicate floppy and rigid blastoderms, respectively, as judged by the GC relative size. ρ Spearman correlation (B, D).

To test whether this could be a mechanism to explain how cell-cell adhesion determines the experimentally observed transitions between floppy and rigid cell connectivity networks within the blastoderm, we determined relative cell-cell tension α across different time points during blastoderm fluidization/thickening by measuring the contact angle $\theta_{e}$ between blastoderm cells at their interface to the interstitial fluid (Figures 4A and 4B; see STAR Methods for determining α). We found an increase in α, and thus a decrease in cell-cell adhesion, during blastoderm fluidization (Figures 4B and B’) and qualitatively similar local cellular connectivity patterns as theoretically predicted (Figure 4A). Moreover, performing the same analysis across multiple time points and experimental conditions (*e-cadherin-MO*, marginal blastoderm, mesoderm induced) (Figure S4A), revealed a close correlation between α and the average connectivity (Figure 4C), GC size (Figure S4B), cell fraction (Figure S4C) as well as tissue viscosity (Figure 4D), suggesting that α is functionally linked to those properties. Remarkably, we also found that blastoderm fluidization occurred for values of α≈0.85−0.9 that are close to the values theoretically predicted by our mechanical model for the transition between floppy and rigid local topologies (Figures 4A and S4A). Likewise, simulating cell-cell contact formation as a function of α for large non-confluent 2D cell aggregates using our mechanical model not only produced cell connectivity maps qualitatively matching our experimental cell-cell connectivity graphs but also showed a floppy-to-rigid network transition for similar α values as found in the 4-cell mechanical model (Figure S4E). Together, this suggests a simple explanation of how alterations in cell-cell adhesion can trigger global cell network topology and rigidity changes, in agreement with the ones observed during blastoderm fluidization/thickening process.

### Meta-synchronous cell cleavages drive uniform tissue fluidization by triggering random cell connectivity changes

**Figure 5:**
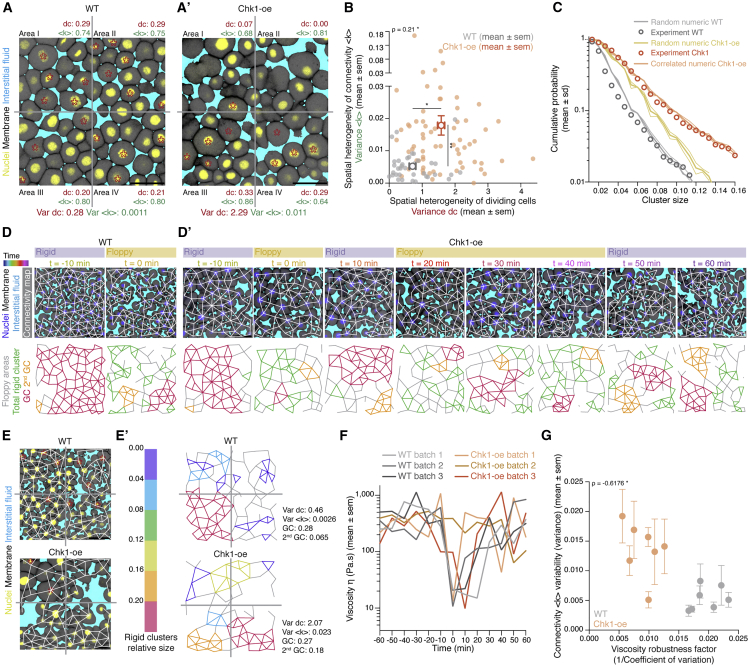
Uniformity in blastoderm rigidity transition relies on meta-synchronous cell divisions generating random cell connectivity changes (A and A’) Exemplary 2D confocal sections at the 1^st^–2^nd^ deep-cell layer of the central blastoderm of a WT (A) and a Chk1-overexpressing (Chk1-oe, A’) embryo during the last round of meta-synchronous cell cleavages. Interstitial fluid is marked by dextran, nuclei by H2B-GFP, and membranes by membrane-RFP. The Var for the fraction of dividing cells (red stars, *dc*) and normalized connectivity <k> was calculated between the quadrants. (B) Plot of the spatial heterogeneity in connectivity as a function of the spatial heterogeneity in the fraction of dividing cells, expressed as the Var in <*k>* and Var in *dc*, respectively, between the quadrants (mean ± SEM) shown in (A) and (A’) (WT n = 36 blastoderms, N = 5 embryos; Chk1-oe n = 66 blastoderms, N = 5 embryos; individual Var values are shown in the overlaid dot plot). (C) Plot of the distribution of rigid cluster sizes p(s) other than the GC for all range of connectivity values in (i) WT and Chk1-oe experimental networks (circles), (ii) simulated random networks with size distribution identical to the experimental ones (WT, shaded gray; Chk1-oe, shaded yellow), and (iii) simulated correlated networks with size distribution identical to the experimental Chk1-oe (shaded orange) (WT n = 103 networks, N = 11 batches, Chk1-oe n = 95 networks, N = 5 batches), showing that Chk1-oe cluster size distribution is wider than the one expected from a model random network, but displays a very good fit if the model network shows spatial correlations in division/bond loss. (D and D’) Exemplary 2D confocal sections at the 1^st^–2^nd^ deep-cell layer of the central blastoderm of a WT (D) and a Chk1-oe (D’) embryo with overlaid connectivity maps (top) and their rigidity profile (bottom) at different time points during the fluidization/thickening process (color coded), marked as in (A). Floppy areas are illustrated in gray, rigid areas in green, the 2^nd^ GC in orange, and the GC in red. Yellow- and purple-shaded areas indicate floppy and rigid blastoderms, respectively, as judged by the GC relative size. (E) Exemplary 2D confocal sections at the 1^st^-2^nd^ deep-cell layer of the central blastoderm of a WT (top) and a Chk1-oe (bottom) embryo, marked as in (D), at a fluidized state with marked mitotic cells (red stars) and overlaid connectivity maps. (E’) Their rigidity profile is color coded for the size of the rigid clusters (fraction occupied in the total network). (F) Plot of blastoderm tissue viscosity as a function of time for measurements from 3 independent embryo batches of WT (showing synchronous fluidization) and Chk1-oe (showing heterogeneous phases of fluidization/thickening) embryos. (G) Plot of normalized connectivity <k> variability, expressed as the Var in normalized connectivity <k> between the quadrants (mean ± SEM) shown in (A) and (A’) as a function of a robustness viscosity factor, expressed as the inverse of coefficient of Var between the viscosity measurements (see Figure S5J) during cell-cycle meta-synchrony. Kruskal-Wallis test (B), ρ Spearman correlation test (B and G). Scale bars: 50 μm in (A), (A’), (D), (D’), and (E). See also Figure S5, Table S1, and Video S3.

Although the blastoderm displays hallmarks of criticality, suggesting that small connectivity changes can already trigger a rigidity PT, the actual changes in blastoderm viscosity are highly uniform, robust, and reproducible (Figure 1C). This implies that the cell network connectivity within the blastoderm must be tightly controlled in development. To obtain insight into these control mechanisms, we turned to the role of the last two rounds of meta-synchronous blastoderm cell cleavage cycles (∼1 h) (Keller et al., 2008; Olivier et al., 2010), which we have previously found to trigger a gradual reduction in cell connectivity (Petridou et al., 2019). Given that cell divisions are randomly and thus homogeneously distributed throughout the blastoderm due to the synchrony of the cleavage cycles (Figure 5A, small variance in the fraction of dividing cells between the quadrants), it is conceivable that synchronous cell divisions are important for uniform tissue fluidization within the blastoderm center. To experimentally address this, we sought to interfere with cell-cycle synchrony in order to create a spatial heterogeneity in the distribution of dividing cells within the blastoderm and, consequently, break the spatial homogeneity of cell contact loss.

**Figure S5:**
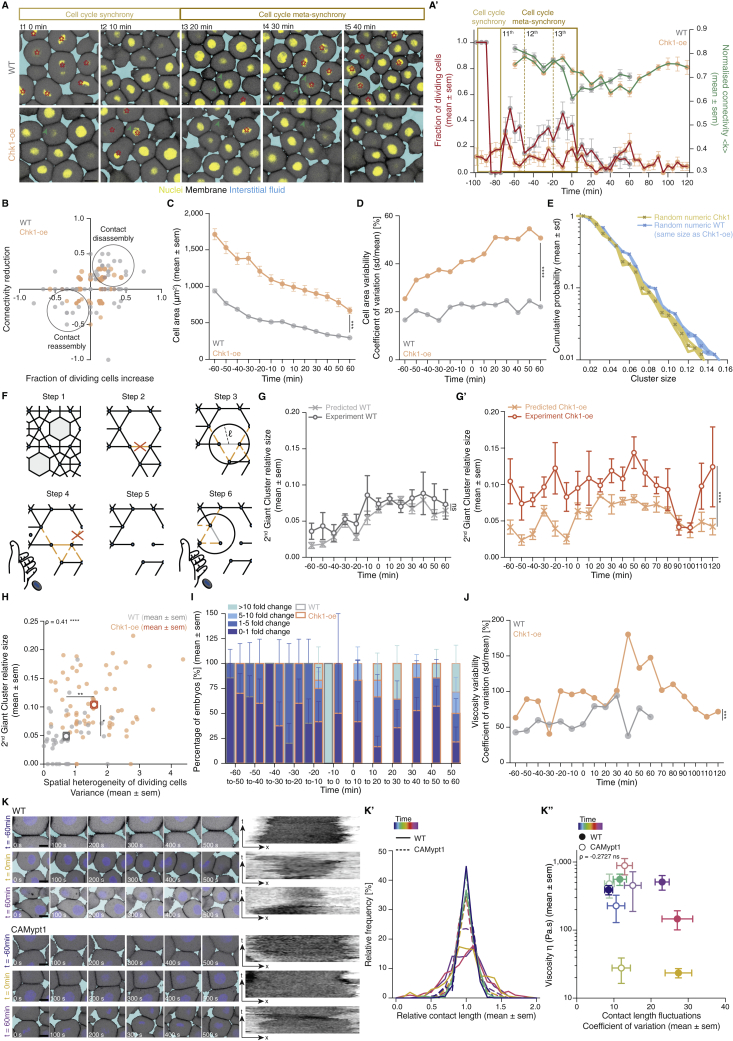
Effects of Chk1 overexpression in cellular, topological, and material properties of the zebrafish blastoderm and role of cell contact length fluctuations on tissue viscosity, related to Figure 5 (**A**) Exemplary high magnification confocal images at consecutive time points during the fluidization/thickening process (10 min interval) of the central blastoderm in WT (top) and Chk1-overexpressing (oe) (bottom) embryos, during synchronous (all the cells in the field of view are dividing at the same time point) and meta-synchronous (only a fraction of the cells is dividing) cell cleavages. Nuclei are marked by H2B-GFP, membranes by membrane-RFP, and interstitial fluid by dextran. Red asterisks indicate mitotic cells, green arrowheads point at contact disassembly in meta-synchronously dividing cells of WT embryos (top) and contact maintenance in asynchronously dividing cells of Chk1-oe embryos (bottom). (**A’**) Plot of the fraction of dividing cells (mean ± sem) (left axis) from 2D confocal sections of WT (n = 10 blastoderm areas per time point, N = 4) and Chk1-oe (n = 8 blastoderm areas per time point, N = 4) embryos as shown in (A), and of normalized connectivity < k > (mean ± sem) (right axis) from 2D confocal sections as shown in Figures 5D and 5D’ for WT (n = 11 networks per time point, N = ) and Chk1-oe (n = 5 networks per time point, N = 5 embryos) embryos, as a function of time during the fluidization/thickening process, lasting until 60 min in WT and 120 min in Chk1-oe embryos (see STAR Methods for timing difference). Dashed lines indicate the duration of the 11^th^, 12^th^and 13^th^meta-synchronous cell cycles in WT. (**B**) Dot plot of the relative reduction in central blastoderm cell connectivity versus the increase in the fraction of dividing cells within 10 min intervals determined on 2D confocal sections of WT (n = 63 blastoderm areas, N = 5 embryos) and Chk1-oe (n = 94 blastoderm areas, N = 5 embryos) embryos as shown in (A) over the time period directly preceding blastoderm fluidization (1h for WT and 2h for Chk1-oe embryos). (**C**) Plot of cell area (mean ± sem) as a function of time during the fluidization/thickening process in WT (n = 652 cells, N = 6 embryos) and Chk1-oe (n = 999 cells, N = 5 embryos) central blastoderms. (**D**) Plot of the cell area variability expressed as coefficient of variation (sd/mean) of the data plotted in (C). (**E**) Numerical check to discard size and connectivity distribution effects in the discrepancy of Chk1-oe cluster size distribution from its expected random counterpart (yellow curve in Figure 5C). The blue curve is obtained from a re-scaled version of the cluster size distribution found in WT experimental networks, in a way that the average size matches the one found in Chk1-oe experimental networks. (**F**) Schematic illustration of the model designed to generate spatially correlated link/bond loss, with correlation length ℓ (at the scale of cell diameter) and correlation probability λ. In step 1, a given state of a lattice of cell contacts is presented. In step 2, a link is chosen at random and removed (orange cross). In step 3, the links in the neighborhood (orange dashed lines) of the removed link (light gray) are identified (black circle), using as a parameter the correlation length ℓ. In step 4, a biased coin is drawn with probability λ to show ‘face’, where ‘face’ removes a link from this neighborhood (orange cross). If ‘tail’ appears, the algorithm goes back to step 2 and the process is restarted. If all the links in the neighborhood are removed (step 5), a biased coin is drawn again (step 6) and if ‘face’ appears, a link that was in the neighborhood is chosen at random and the algorithm goes back to step 3, now taking this randomly chosen link as the starting point. If ‘tail’ appears, the algorithm goes back to step 2. The process starts with a complete lattice and ends when the average connectivity of the networked to be simulated is achieved (see STAR Methods for details). (**G-G’**) Plots of the fraction of the network occupied by the 2^nd^ Giant Cluster (GC) (mean ± sem) in simulated and experimental networks of WT (**G**) and Chk1-oe (**G’**) central blastoderms as a function of time during the fluidization/thickening process (WT simulated and experimental, n = 103 networks, N = 11 embryos, each; Chk1-oe simulated and experimental, n = 95 networks, N = 5 embryos). (**H**) Plot of the size of the 2^nd^GC (mean ± sem) as a function of the spatial heterogeneity in the fraction of dividing cells (expressed as the variance in the fraction of dividing cells between the quadrants shown in Figure 5A, mean ± sem) in WT and Chk1-oe central blastoderms (WT n = 36 blastoderms, N = 5 embryos; Chk1-oe n = 66 blastoderms, N = 5 embryos; individual blastoderm values are shown in the overlaid dot plot). (**I**) Plot of the percentage of central blastoderms from different embryo batches undergoing the indicated fold-changes in their viscosity within 10min intervals during the fluidization/thickening process for WT (n = embryos, N = 8 batches) and Chk1-oe (n = embryos, N = 9 batches) embryos. (**J**) Plot of the central blastoderm viscosity variability expressed as coefficient of variation (sd/mean) from viscosity measurements of 8 independent WT or Chk1-oe embryo batches. (**K**) Exemplary time stills (left) from high magnification confocal time series of cell-cell contact dynamics in WT (top) and CAMypt1 expressing embryos (bottom) and corresponding kymograph (right) during the fluidization/thickening process. (**K’**) Plot of the relative frequency of cell-cell contact lengths in WT (solid line) and CAMypt1 expressing embryos (dashed line) during the fluidization/thickening process (color coded for 30 min intervals) (n = 10 contacts, N = 4 embryos for each experimental condition and time point). (**K’’**) Plot of viscosity values (mean ± sem) as a function of contact length fluctuations expressed as coefficient of variation (sd/mean) from the average contact length over a 10 min period in WT (filled circle) and CAMypt1 (clear circle) expressing embryos during the fluidization/thickening process (color coded for 30 min intervals) (for contact length fluctuation: n = 10 contacts, N = 4 embryos for each experimental condition and time point; for viscosity: WT, n = 53, N = 11; CAMypt1, n = 27, N = 7; n, number of embryos, N, number of embryo batches). Kruskal-Wallis test (G’, H), Mann-Whitney test (C, D, J), ρ Spearman correlation (H, K’’). Scale bars, 20 μm (A) 10 μm (K).

To this end, we overexpressed Chk1, previously shown to slow down the cleavage cycle oscillator (Zhang et al., 2014), and we found that it also reduced cell-cycle synchrony (Figures S5A–S5D), leading to an increase in the heterogeneity of the spatial distribution of dividing cells within the blastoderm (Figure 5A’). Consequently, Chk1-overexpressing embryos showed a highly heterogeneous spatial connectivity profile within the blastoderm (Figures 5A’ and 5B), altering its structural properties (Figures 5C–5E). The latter was evidenced by a broader rigid cluster size distribution than observed in WT embryos and theoretically expected for random networks of the same size (Figures 5C and S5E; see STAR Methods). However, by incorporating local correlations in links/adhesion in our simulations to account for the experimentally observed heterogeneous spatial connectivity profile (Figure S5F; STAR Methods), we were able to closely recapitulate the cluster size distribution in Chk1-overexpressing embryos (Figure 5C, orange-shaded line versus orange circles). Moreover, consistent with predictions from those simulations, we experimentally observed that the size of the 2^nd^ biggest cluster after the GC (Figures S5G and S5G’; Figure 5D, orange cluster; Videos S2 and S3) was much bigger in Chk1-overexpressing embryos than in WT networks (Figures S5G and S5G’). This size increase partially scaled with the degree of heterogeneity in the spatial distribution of dividing cells (Figure S5H), thereby directly linking the randomness in cell divisions, and thus cell-cycle synchrony, to the structural properties of the blastoderm.

Finally, we made use of Chk1-overexpressing embryos to assess how a heterogeneous spatial connectivity profile within the blastoderm affects the robustness of blastoderm fluidization. To this end, we measured tissue viscosity in Chk1-overexpressing embryos throughout the period of blastoderm fluidization and thickening in WT embryos. Strikingly, we found that the blastoderm in Chk1-overexpressing embryos did not show one distinct fluidization and thickening cycle, as seen in WT embryos, but instead went through several cycles of partial fluidization and thickening (Figures 5F and S5I), in agreement with the observation that the GC in these embryos appears and disappears several times (Figure 5D’; Video S3). Moreover, the variation in viscosity values obtained at distinct time points during the fluidization/thickening process was considerably higher in Chk1-overexpressing compared to WT embryos (Figure S5J). Strikingly, when comparing the variation in blastoderm viscosity to the heterogeneity in the connectivity of these embryos during the meta-synchronous cell cleavages, we detected a clear anti-correlation between these two features (Figure 5G). Collectively, these findings suggest that cell cleavage synchrony serves as an effective mechanism for uniform cell connectivity changes within the blastoderm that is required for uniform and robust blastoderm fluidization.

Video S3. Analysis of temporal changes of central blastoderm rigidity in Chk1-overexpressing embryos using rigidity percolation, related to Figure 5 Exemplary time lapse video (4.5 min interval) of the central blastoderm of a Chk1-overexpressing embryo with overlaid connectivity maps (left) and rigidity analysis (right). Nuclei are marked by H2B-GFP, membranes by membrane-RFP and interstitial fluid by dextran. Floppy areas are illustrated in gray, rigid areas in green, the Giant Cluster (GC) in red and the 2^nd^ GC in orange. Rigid time points are marked in purple, floppy time points in yellow. Scale bar, 50 μm.

## Discussion

Our findings demonstrate that the zebrafish blastoderm undergoes a genuine rigidity PT and provides mechanistic insight into the structural origin and developmental regulation of this PT within the organism.

The major challenge in probing the structural basis of a PT—which we have addressed in this study—is to link microscopic control parameters to macroscopic rheological changes, an approach requiring combined measurements of local cell behavior with direct tissue-scale rheological measurements. In previous studies on confluent monolayers, material properties were indirectly inferred from a simple criterion derived from vertex or Voronoi models, the normalized cell perimeter (which coarse-grains details of the cell contact mechanics; Bi et al., 2015; Farhadifar et al., 2007; Krajnc et al., 2018; Merkel and Manning, 2018; Popović et al., 2020), as direct rheological measurements were difficult (Angelini et al., 2011; Garcia et al., 2015; Miroshnikova et al., 2018; Park et al., 2015; Tetley et al., 2019), hampering the characterization of a bona fide PT in those tissues. Recent *in vivo* studies in zebrafish have managed to probe tissue fluidization during development, specifically within the presomitic mesoderm (PSM) and early blastoderm, via direct rheological measurements (Mongera et al., 2018; Petridou et al., 2019), allowing to experimentally probe tissue PTs. However, a theoretical framework for modeling and inferring PTs in such non-confluent tissues was lacking. By applying rigidity percolation, a theoretical concept widely used in material science, to biological tissues, we provide a simple criterion readily testable in any non-confluent tissue, without requiring knowledge of the detailed microscopic properties (which are coarse-grained within the cell-cell contact topology). Importantly, the zebrafish blastoderm provides a system in which a direct rheological transition can be monitored in both space and time, a key prerequisite for testing our framework and pinpointing hallmarks of PTs. We show that rigidity percolation accurately predicts the material properties of the system throughout morphogenesis as well as across multiple experimental perturbations of cell fate, division, contractility, and adhesion. Furthermore, we demonstrate hallmarks of criticality *in vivo*, such as power-law exponents and its associated discontinuities of macroscopic observables at criticality. Given that those hallmarks have not yet been clearly demonstrated in biological settings—mostly due to difficulties in parameter fine-tuning, measurement precision, and finite size statistics—their identification provides additional strong support for biological tissues undergoing material PTs.

Our findings suggest that analyzing cell-cell contact topology is sufficient to explain tissue PTs during morphogenesis. But how is this coarse-grained observable mechanistically regulated? For instance, our MPA experiments indicate that the blastoderm behaves largely as a simple viscous fluid, while classical rigidity percolation of spring networks provides information about elasticity. We have addressed this apparent discrepancy by showing that viscous networks, where contacts can break and heal, qualitatively change their response to external deformations at the same critical point of connectivity as elastic ones. This suggests that using rigidity percolation theory is appropriate to describe the viscosity changes observed within the blastoderm, although quantitative understanding of the absolute value of viscosity will require more detailed modeling of the nonlinear and time-dependent properties of the cell contacts. Indeed, for the jamming of sphere packings, spring network models, such as the ones we used, can only predict the critical point and linear response upon shear (van Hecke, 2010), while other responses such as bulk modulus or nonlinear rheology would require further extension of the theory to include mechanical elements such as re-arrangements (David et al., 2014), cell-fluid interaction (Recho et al., 2019), and cell-cell friction (Liu et al., 2019). The influence of adhesion, for instance, has been probed experimentally using a biomimetic system of attractive emulsion droplets (Jorjadze et al., 2011; Pontani et al., 2012). Adhesion is theoretically predicted to markedly change the nature of the jamming transition, leading to a slow growth of the rigid cluster as a function of sphere fraction around criticality (Koeze and Tighe, 2018; Lois et al., 2008), closely matching our findings (Figure S4D).

Interestingly, decreasing adhesion in 2D vertex models of fully confluent tissues has been shown to increase tissue rigidity (Bi et al., 2015). However, such an effect would only apply up to the point where loss of adhesion breaks tissue confluency and where cell-cell connectivity then becomes key. To gain insight into this process, we have analyzed the microscopic basis of this framework, via a model of cell-cell contacts arising from minimization of cell-cell and cell-fluid surface energy, suggesting that changing the ratio of both tensions (absolute values of adhesion) is enough to transit between rigid and floppy network configurations. Similarly, Kim et al. (2020) have extended the vertex model to incorporate fluid pockets, cell-cell adhesion, and active fluctuations in order to describe a fluidization transition within the PSM, as assessed via local micro-rheology and a caged-to-diffusive transition in the MSRD of cells. This fluidization transition in PSM has further been proposed to arise from changes in cell-cell tension/adhesion fluctuations, together with some changes in the absolute values of these tensions influencing cell-cell connectivity. Intriguingly, our functional experiments, where MSRD and cell-cell tension/adhesion fluctuations were reduced by partial inhibition of myosin II activity without affecting cell-cell connectivity changes (Figures S3G and S3J), showed no effects on rheological tissue properties (Figures S3I, I’, S3J, S5K, and S5K’’), consistent with the observation in WT embryos that contact fluctuations do not correlate well with changes in blastoderm tissue viscosity (Figures S1B and S5K). This suggests that the specific cellular properties controlling tissue PTs might be highly context dependent, while the framework of rigidity percolation can provide generic insights into material properties in a parameter-free manner based on a single coarse-grained measurable observable. More generally, applying the concept of rigidity percolation to biological tissues allows importing key concepts and the multi-faceted toolbox of statistical physics to understand the universal features of tissue material properties, although the specific properties of different tissues are likely, as in material science, to depend on different microscopic parameters within this framework.

Finally, being close to a PT has been proposed to be advantageous (Krotov et al., 2014), allowing a tissue to change its material properties rapidly and drastically. However, it also might pose a risk as the tissue, when placed close to criticality, could ectopically change its material properties due to noise. To avoid ectopic PT, cell-cell contact loss must therefore be tightly controlled in space and time. Our findings suggest that the meta-synchrony of the cell cycle provides such control mechanisms, where largely synchronous cell divisions lead to random and thus homogeneously distributed contact loss throughout the tissue. This is consistent with previous findings that have linked cell divisions to tissue fluidity (Firmino et al., 2016; Petridou et al., 2019; Ranft et al., 2010; Saadaoui et al., 2020). We also show that cellular fate modulates cell-cell connectivity and thus distance to the critical point, hinting at an intricate relationship between cell division, fate specification, and tissue material properties in development, with potential application in pathological processes such as tumor invasion (Douezan et al., 2011; Gonzalez-Rodriguez et al., 2012; Haeger et al., 2014; Hannezo and Heisenberg, 2019; Oswald et al., 2017).

### Limitations of study

In our pipette aspiration assays (Figure 1B), we have restricted ourselves to the study of the regime where the deformation distance grows linearly in time (consistent with a simple Newtonian fluid), which allows us to extract a unique rheological quantity, i.e., viscosity (Figure 1C). In the future, it would be important to probe different regimes systematically with different pipette aspiration pressures, to understand potentially more complex rheological behaviors, such as yield stress or viscoelasticity. Moreover, while we have shown that the transition in rheological property of the blastoderm is well predicted by rigidity percolation (Figure 1H), a more quantitative understanding on what sets the absolute values of rheological quantities would require an extension of the model, taking into account cellular shape but also the mechanics of cell contacts, including frictional dissipation and re-arrangements. To complement this experimentally, it would be important to further qualify what constitutes a cell-cell contact. Here, we have relied on a binary definition (presence or absence of interstitial fluid between two membranes; see Figure 2A) and have assessed average contact strength at a given time point via junctional angle measurements (Figure 4), but studying the heterogeneities of contact strength both in time and space might help in better understanding the overall tissue material properties.

## STAR★Methods

### Key resources table

**Table:** 

REAGENT or RESOURCE	SOURCE	IDENTIFIER
**Antibodies**		

Anti-E-Cadherin (zebrafish) rabbit Antibody	Schwayer et al., 2019	N/A
Alexa Fluor 546 goat anti-rabbit IgG (H+L)	Thermo Fisher Scientific	Cat# A-11010; RRID: AB_2534077

**Chemicals, peptides, and recombinant proteins**		

Alexa Fluor 647 Dextran 10,000 MW	Invitrogen	Cat# D22914

**Experimental models: Organisms/strains**		

Zebrafish: AB wild-type	MPI-CBG Dresden	N/A
Zebrafish: *slb* / MZ *wnt11 f2*	Heisenberg et al., 2000	N/A

**Oligonucleotides**		

Control MO. 5′-ATGCCAGAGTTCTTACAGAAGCGAT-3′	Barone et al., 2017	N/A
E-cadherin MO: 5′-TAAATCGCAGCTCTTCCTTCCAACG-3′	Babb and Marrs, 2004	N/A
Casanova MO: 5′-GCATCCGGTCGAGATACATGCTGTT-3′	Krieg et al., 2008	N/A

**Recombinant DNA**		

pCS2-H2BGFP	Keller et al., 2008	N/A
pCS2-membraneRFP	Iioka et al., 2004	N/A
pCS2-CAMypt1	Schwayer et al., 2019	N/A
pCS2-Chk1	Shamipour et al., 2019	N/A
pCS2-ndr2 cyclops	Krieg et al., 2008	N/A

**Software and algorithms**		

Fiji	Schindelin et al., 2012	https://fiji.sc
Imaris	Bitplane	https://imaris.oxinst.com/packages
GraphPad Prism	GraphPad Software	https://www.graphpad.com/scientific-software/prism/
The Surface Evolver	Brakke, 1992	http://facstaff.susqu.edu/brakke/evolver/evolver.html
The Pebble game	Jacobs and Thorpe, 1995; Zhang et al., 2015	https://github.com/coldlaugh/pebble-game-algorithm/blob/master/pebble.pyx
Micropipette Assay measurement script	Petridou et al., 2019	https://github.com/bernatze/Lattice_Deformation
Deformation of elastic/viscous networks	This study	https://github.com/bernatze/Lattice_Deformation

### Resource availability

#### Lead contact

Further information and requests for resources and reagents should be directed to and will be fulfilled by the Lead Contact, Carl-Phillip Heisenberg (heisenberg@ist.ac.at).

#### Materials availability

This study did not generate new unique reagents.

#### Data and code availability

The Fiji macro used to quantify the MPA experiments and the codes used for the rigidity simulations, correlation model and the deformation of elastic/viscous networks are available at https://github.com/bernatze/Lattice_Deformation.

### Experimental model and subject details

Zebrafish (*Danio rerio*) were maintained under a 14 h light/10 h dark cycle (Detrich et al., 1999). The following zebrafish strains were used in this study: wild-type (WT) AB and *slb*/*wnt11 f2* (Heisenberg et al., 2000). Zebrafish embryos were grown at 25-28.5°C in E3 embryo medium and staged as previously described (Kimmel and Law, 1985). For precise staging before and during blastoderm spreading, the last rounds of meta-synchronous cleavages and resulting changes in cell size were used as temporal hallmarks defining developmental time relative to the onset of doming. Chk1-overexpressing (oe) embryos displayed approximately 1 h of developmental delay, thus we extended their analysis by 1 h. Onset of doming (t = 0 min) was defined as the time when during the viscosity measurements the first embryo within an egg-lay/batch underwent fluidization. All animal experiments were carried out according to the guidelines of the Ethics and Animal Welfare Committee (ETK) in Austria.

### Method details

#### Embryo microinjections

Zebrafish embryos were injected using glass capillary needles (30-0020, Harvard Apparatus, MA, USA), which were pulled by a needle puller (P-97, Sutter Instrument) and attached to a microinjector system (PV820, World Precision Instruments). Microinjections of mRNAs and *morpholinos* (MOs) were performed at the one-cell stage. mRNAs were synthesized using mMACHINE SP6 kit (Ambion). The following mRNAs were injected: 70 pg *membrane RFP* (Iioka et al., 2004), 70 pg *H2B GFP* (Keller et al., 2008), 200 pg *CAMypt1* (Schwayer et al., 2019), and 72 pg *chk1* (Shamipour et al., 2019). The following MOs were injected: 4 ng *e-cadherin* MO, 4 ng *control* MO for *e-cadherin* (Babb and Marrs, 2004). To induce mesodermal progenitor cell fate, the following combination of mRNAs and MOs was injected: 100 pg *ndr2l cyclops* mRNA and 2 ng *casanova* MO (Krieg et al., 2008). To label the interstitial fluid, 1 nL of 0.6 mg/ml dextran Alexa Fluor 647 (10,000 MW; D22914, Invitrogen) was injected in the blastoderm of 1k-stage embryos (∼3 hpf).

#### Micropipette aspiration and viscosity measurements

Blastoderm viscosity was measured by micropipette aspiration based on previously established protocols (Guevorkian et al., 2010; Petridou et al., 2019). Briefly, embryos were placed on 3% methylcellulose coated glass coverslips in 1x Danieau’s solution on an inverted Leica SP5 microscope. A fire-polished, passivated (with heat inactivated FBS) micropipette of 35 μm inner diameter, 30° bent, with a spike end (Biomedical Instruments) was inserted into the blastoderm center or margin, just below the EVL. The micropipette movements were controlled by motorized micromanipulators (Eppendorf Transferman, Nk2). Upon insertion of the pipette in the blastoderm, an aspiration pressure of 150 Pa was immediately applied using a Microfluidic Flow Control System Pump (Fluigent, Fluiwell) (with negative pressure ranging from 7-750 Pa, a pressure accuracy of 7 Pa and change rate of 200 Pa.s^-1^) and the Dikeria micromanipulation software. The value of the applied pressure for the measurements at all stages during blastoderm fluidization/re-solidification (150 Pa) was set according to prior test aspiration experiments, where the applied pressure was continuously increased in a stepwise process (10 Pa / 20 s) until the aspirated tissue started flowing into the pipette. For creep and recovery experiments, pressure was applied until the tissue flew into the pipette at a constant velocity (for ∼3 min, except the cases where the tissue was fluidized and the deformation was too fast) and then pressure was immediately released. Constant tissue flow velocity was similar for aspiration times of 3, 4, 5, and 6 min, and experiments were thus performed with 3min aspiration time (Figure S1A). Images for monitoring the aspiration and relaxation of the tissue were acquired every 500 ms. Viscosity calculations were performed as previously described (Guevorkian et al., 2010; Petridou et al., 2019). Briefly, the tongue length for each time point was measured using a customized Fiji macro, and changes in tongue length during aspiration and relaxation were then plotted over time (Figure 1B). The slope of the aspiration curve at the point of constant flow depends on the viscosity η, $L_{asp}=\frac{R_{p}\;\left(\text{Δ}P-\;P_{c}\right)}{3\pi \eta }\;$ with R being the radius of the pipette, ΔP the applied pressure and P_c_ the critical pressure. When the pressure is set to zero during the relaxation, the tissue retracts at a velocity $L_{ret}=\frac{R_{p}\;\left(\;P_{c}\right)}{3\pi \eta }\;$. From the aspiration and retraction rates, viscosity can be calculated as $\eta =\;\frac{R_{p}\text{Δ}P\;}{3\pi \left(L_{asp}+L_{ret}\right)}$. In case the retraction rates are very low, then the major determinant of viscosity is the aspiration rate.

#### Immunostaining

Embryos were fixed in 2% paraformaldehyde (PFA) for 4 h at RT. After fixation, embryos were washed in 0.5% Tween-20 (in 1x PBS) overnight at 4°C, dechorionated and washed in 0.5% Tween-20, 0.5% Triton X-100, 0.1 M glycine (in 1 x PBS) for 1 h at RT. Embryos were then incubated in blocking solution (0.5% Tween-20, 0.5% Triton X-100, 1% DMSO, 1% BSA in 1 x PBS) for 3-4 h at RT and then incubated with primary antibody (rabbit E-cadherin anti-zebrafish (Petridou et al., 2019), 1:200, generated at MPI-CBG) diluted in blocking solution overnight at 4°C. Embryos were subsequently washed 4 × 20 min in 0.5% Tween-20 and incubated with secondary antibody (goat Alexa Fluor 546 anti-rabbit, 1:500, A11010 ThermoFisher Scientific) diluted in blocking solution overnight at 4°C. Finally, embryos were washed 4 × 20 min in 0.5% Tween-20, post-fixed in 4% PFA for 30 min at RT and imaged (Figure S3B).

#### *In situ* hybridization

*In situ* hybridization was performed as previously described (Thisse et al., 1994). Briefly, 4.3 hpf embryos were fixed in 4% PFA overnight and then dehydrated and stored for at least 1 day in 100% Methanol at – 20°C. Embryos were then rehydrated, permeabilized and incubated in hybridization buffer and with the RNA probes overnight. Embryos were washed in serial dilutions of SSC, incubated with an Alkaline phosphatase anti- digoxigenin (DIG) antibody overnight (A2237, Sigma), then washed and stained with a mixture of 4-Nitro blue tetrazolium chloride (NBT) (11383221001, Roche) and 5-brome-4-chloro-indolyl-phosphate (BCIP) (11383221001, Roche). Antisense RNA probes were synthesized from cDNA for *ntl*, *gsc* and *wnt11* (Heisenberg et al., 2000), using SP6, T7 or T3 RNA polymerase from mMessage mMachine kits (AM1344, ThermoFisher) with DIG RNA-labeling kit (11 277 073 910, Sigma) (Figure S3D).

#### Image acquisition

Dechorionated embryos were mounted in 0.5% low melting point agarose (16,520-050, Invitrogen) on a glass bottom dish (P35G-1.5-14-C, MatTek Corporation). Mounted embryos were kept in an incubation chamber at 28.5°C throughout acquisition. Whole embryo single plane bright-field/fluorescence imaging was performed on a Nikon Eclipse inverted wide-field microscope equipped with CFI Plan Fluor 10x/0.3 objective (Nikon) and a fluorescent light source (Lumencor). For high magnification confocal imaging of deep cells to reconstruct connectivity maps, a Zeiss LSM880 inverted microscope, equipped with a Plan-Apochromat 40x/NA 1.2 water-immersion objective (Zeiss), GaAsP and multialkali PMT detectors was used. The Fast Airyscan super-resolution module was used for high magnification confocal imaging of cell-cell contact length fluctuations. For imaging the micropipette aspiration experiments, a Leica SP5 inverted microscope equipped with a resonant scanner and a HC Plan-Apochromat 10x/NA 0.4 objective (Leica) was used. For imaging the immunofluorescence experiments on E-cadherin levels, a Zeiss LSM880 upright microscope, equipped with a Plan-Apochromat 20x/1.0 water immersion DIC objective was used. For imaging the *in situ* hybridization experiments, an Olympus SZX 12 stereo-microscope, equipped with a QImaging Micropublisher 5.0 camera was used.

#### Reconstruction of connectivity maps and rigidity analysis

Cell connectivity was defined on 2D confocal sections of the 1^st^-2^nd^ or 3^rd^-4^th^ deep-cell layer, in which the nuclei, cell–cell contacts and interstitial fluid accumulations were differentially labeled. The image from the interstitial fluid channel was converted to a binary image and processed by a median filter of 2-pixel width in Fiji, to emphasize the interstitial fluid gaps. Cell nuclei of cells within the same focal plane that had no interstitial fluid between them were considered as contacting cells. By using the multi-point tool in Fiji, the coordinates of each nuclei and their contacting-neighbors were marked and plotted using standard python package matplotlib. Due to the manual identification of connections, the connectivity of each image was corrected twice by overlaying the connectivity map on top of the blastoderm image. The rigidity analysis to identify floppy and rigid areas, Giant and 2^nd^ Giant clusters, and cluster size distributions were performed on the connectivity maps using a python version of the pebble algorithm pebble.py (Key resources table).

#### Numerical construction of model networks

In this section we present the precise method used to generate the lattices that serve as a model of the cell contact networks analyzed in this study.

We worked in general with random triangular lattices, G(V,E), embedded in a flat 2D space (see Figure S1H). Throughout the text, we will refer indistinctly to them as *networks* or *lattices*. $V=\left\{v_{1},...,v_{N}\right\}$ is the set of N nodes and $E=\left\{e_{1},...,e_{M}\right\}$ is

the set of M links connecting nodes of V. We call average connectivity ⟨C⟩ the average number of edges connected to a given node, defined as:

$$\left\langle C\right\rangle =\frac{2M}{N}.$$

A fully connected triangular lattice contains all possible links between the nodes, and is typically used to model packing problems (Jacobs and Hendrickson, 1997; Jacobs and Thorpe, 1995, 1996). For triangular 2D lattices, in the limit N→∞, ${\left\langle C\right\rangle }_{Max,N}\to 6$. For finite lattices, the low number of links connecting the nodes in the boundary implies a reduction of this maximum connectivity, ${\left\langle C\right\rangle }_{Max,N}<6$, and the specific number will depend on the exact value of N and the node arrangements. Considering that nodes are arranged in a square with sides $L∼\sqrt{N}$ (corresponding to the shape of experimental networks), ${\left\langle C\right\rangle }_{Max,N}$ can be very well approximated as:

$${\left\langle C\right\rangle }_{Max,N}∼\frac{8+16\left(L-2\right)+{6\left(L-2\right)}^{2}}{N}.$$

Square geometries are commonly used in the study of rigidity percolation (Jacobs and Thorpe, 1995, 1996), where finite size effects are well understood (Goodrich et al., 2013). Our data fits satisfactorily with the square-geometry assumption, although an exception is found in the networks belonging to the margins, since, in these networks, nodes are disposed along irregular geometries. We therefore focused our rigidity analysis to the networks located in the center, since for them we could construct model networks with a unified criterion, thereby reducing the potential effects of the boundaries and enabling fair comparison. As discussed in the main text, we restricted the rigidity analysis to 2D networks, which are more tractable both experimentally and computationally (Chubynsky and Thorpe, 2007). However, the 2D connectivity of the embryo was identical at different z-slices (Figures S1K and S1K’), meaning that no anisotropy in the packing is expected, and that one expects to be able to predict 3D average connectivity from these 2D sections. To test this more directly, we have also measured the local average number of contacts per cell both in 2D and 3D for a subset of randomly selected cells (see Figure S1L’ for further details on the methods of measurement), and confirmed that we also observed a drastic decrease of average connectivity ⟨k⟩ in 3D measurements at the fluidization point (Figure S1L). As expected for triangular lattices, the average number of contacts in 3D was around the double of the average number of contacts in 2D at all time points. Further extension to 3D would require a closer quantification of the exact structure of 3D connectivity, and in particular the type of packing of the blastoderm cells in 3D, which would impact on the threshold. In addition, in order to compare systems with different cellular numbers (which increases as a function of time due to division in the blastoderm), we worked instead with the *normalized connectivity* of a given finite lattice G(V,E) ⟨k⟩ defined as:

$$\left\langle k\right\rangle =\frac{\left\langle C\right\rangle }{{\left\langle C\right\rangle }_{Max,N}}.$$

⟨k⟩ corresponds to the probability that a link exists in the triangular lattice of a given size. In Figure S1H we provide an example of a fully connected lattice and a lattice with average connectivity 3.6.

#### Numerical construction of random triangular lattices

The methods described here are implemented in the python package Lattice.py. In brief, *in silico* random triangular lattices are constructed based on two parameters L and ⟨C⟩. The construction steps are:

• Start from a fully connected triangular lattice made of $∼L^{2}$ nodes arranged uniformly in a square with links of equal length $l_{0}=1$
• Remove links at random until the average connectivity value ⟨C⟩ is achieved.

#### Numerical construction of correlated triangular lattices

In the study of Chk1 overexpression (oe) tissues, we needed to generate spatially correlated lattices, implying that the presence/absence of links is not purely random throughout the tissue, but spatially correlated. *In silico* correlated triangular lattices are constructed following four parameters: L, ⟨C⟩, ℓ and λ. ℓ is the correlation length, which we fixed to be $\ell =l_{0}=1$ a.u. λ∈[0,1] is the parameter accounting for the strength of spatial correlations. Before starting the description of the algorithm, we defined the *vicinity* of a link e as all the links whose middle point is located at a distance ≤ℓ of the middle point of e. The construction steps are:

• Start from a fully connected triangular lattice made of $∼L^{2}$ nodes uniformly arranged in a square with links of equal length
• Choose a link at random and remove it. Call this link e.
• With probability λ, choose a link in the vicinity of the previously chosen link e and repeat the step. With probability 1−λ go to the previous step.
• If the links in the vicinity of e have been all removed, randomly chose a link $e^{\prime }$ from those that have been removed in the vicinity of e, call it $e=e^{\prime }$ and go to the previous step.
• keep the process running until the average connectivity value ⟨C⟩ is achieved.

Effectively, this process removes links sequentially creating *holes* in some areas of the network while leaving other areas highly dense. *Holes* represent sets of links that have been sequentially removed, and its average size follows a Poisson statistics with average *hole* size of order 1/(1−λ). In Figure S5F the method is schematically outlined. As shown in Figures 5C and S5E, this reproduced well the network statistics observed in Chk1 oe embryos, while random networks reproduced well the network statistics of WT embryos. From a biological perspective, such correlations are expected from heterogeneities in cell divisions, which are linked to cell contact loss (Petridou et al., 2019). Given that cells mix relatively little during early morphogenesis, this means that some regions of the embryo will have clusters of “delayed” cells sharing a common ancestor, and which will remove their bonds in a correlated manner, given the experimentally determined correlation between cell division and contact loss.

#### Generic rigidity analysis

The main theoretical concept underlying the present study is *rigidity*. Rigidity is a property related only to the connectivity pattern of the nodes (i.e., it is a *topological* property), but it has direct impact on the material response of the net. Rigidity is a property of a subgraph of a given network, and this is why we refer to *rigid clusters*. Following (Jacobs and Thorpe, 1995), a rigid cluster is defined as “A collection of nodes form a rigid cluster when no relative motion within that cluster can be achieved without a cost in energy.”

In that context, energy costs arise from the stretching or compression of links after a node rearrangement. We will consider only maximal rigid clusters: Given a lattice G(V,E), a subgraph $G^{\prime }\left(V^{\prime },E^{\prime }\right)\subseteq G\left(V,E\right)$ is a *maximal* rigid cluster if i) it is rigid, according to the above definition, and ii) there is no subgraph of G(V,E) that is rigid and contains properly $G^{\prime }\left(V^{\prime },E^{\prime }\right)$. In other words, there is no $G^{\prime \prime }\left(V^{\prime \prime },E^{\prime \prime }\right)\subseteq G\left(V,E\right)$ such that $G^{\prime }\left(V^{\prime }E^{\prime }\right)\subset G^{\prime \prime }\left(V^{\prime \prime },E^{\prime \prime }\right)$ and $G^{\prime \prime }\left(V^{\prime \prime },E^{\prime \prime }\right)$ is rigid. In addition, we consider that clusters are *induced* subgraphs of a given lattice G(V,E). An *induced subgraph* is a subgraph $G^{\prime }\left(V^{\prime },E^{\prime }\right)\subseteq G\left(V,E\right)$ if all the connections between the nodes $V^{\prime }$ present in G are also present in $G^{\prime }$. Therefore, we take this working definition for our purposes and, for the sake of clarity, adapt all the theory to the framework of 2D triangular lattices:

Given a triangular lattice G(V,E) over a 2D plane, a rigid cluster is a maximally induced subgraph $G^{\prime }\left(V^{\prime },E^{\prime }\right)\subseteq G\left(V,E\right)$ by which any movement over the plane of any proper subset of nodes of it will unavoidably result into a stretching or compression of at least one link.

Importantly, the above definition does not exclude i) the presence of more than one rigid cluster in a network – in general, there is a number of them– and ii) the fact that a node participates in more than one rigid cluster. Rigid clusters as defined above are identified using the *Pebble game* algorithm.

As pointed out above, a triangular lattice G(V,E) may contain an arbitrary number of rigid clusters, constrained only by the fact that the minimum number of nodes for a rigid cluster is 3, as a triangle configuration is the minimal rigid structure. If we name $R^{\prime }\left(V^{\prime },E^{\prime }\right)\subseteq G\left(V,E\right)$ a given rigid cluster, we define the *set of all rigid clusters of* G as ***R*** = {*R*_*1*_(*V*_*1*_,*E*_*1*_*)*, …,*R*_*k*_(*V*_*k*_,*E*_*k*_*)*}. Given a cluster $R_{k}\left(V_{k},E_{k}\right)$, its *size* is the number of nodes it contains $S_{k}=\left|V_{k}\right|.$

A particularly relevant element of the set ***R*** is the *largest* or *giant* rigid cluster, $GC^{G}$, of the lattice, due its prominent role in the material response of the network (Koeze and Tighe, 2018). Since the size of the $GC^{G}$ may diverge with the size of the lattice, the most relevant quantity is the relative size of the giant cluster $gc^{G}$ relative to the total number of nodes of the whole lattice, N:

$$gc^{G}=\frac{S_{gc^{G}}}{N}.$$

The second largest rigid cluster, which we refer to as the *second giant cluster* (also referred to simply as second cluster in the text), also plays an important role in our analysis (in particular to identify spatial correlations and heterogeneities in connectivity patterns, as discussed in Figures 5D, 5D’, 5E, S5G, S5G’, and S5H), but it is less relevant in order to understand the material response of the system.

#### Generic rigidity phase transition for triangular lattices

At low/mid values of connectivity, the expected fraction of nodes belonging to the largest rigid cluster is negligible. However, when the connectivity crosses a critical value, the size of the largest cluster experiences an abrupt jump from the previous negligible value to almost all the network. This phenomenon is a second order phase transition and has, as an order parameter, the size of the largest rigid cluster and, as a control parameter the connectivity (Jacobs and Thorpe, 1995; Maxwell, 1864; Moukarzel and Duxbury, 1999). This phase transition has important consequences on the rheological properties of the network.

Specifically, in the limit of infinite triangular lattices, the value of $gc^{G}$ tends to 0 for any value 0≤⟨k⟩<2/3. For ⟨k⟩>2/3, $gc^{G}$ experiences a sharp growth close to 2/3 until it saturates at $gc^{G}=1$. We call $\left\langle k_{c}\right\rangle =2/3$ the critical point of the rigidity phase transition for triangular networks, also known as the isostatic point for rigidity (Jacobs and Thorpe, 1995; Maxwell, 1864). At the critical point, we observed the emergence of a rigid cluster that spans a finite fraction of the lattice in a second order phase transition (Jacobs and Thorpe, 1995; Moukarzel and Duxbury, 1999). Standard analysis of signatures of criticality are based on the study of the behavior of the correlation length ξ close to the critical point, showing a scaling behavior $\xi ∼|\left\langle k\right\rangle -\left\langle k_{c}\right\rangle {|}^{\nu }$, with ν∼1.16.

#### Signatures of criticality in experimental data

Finding traces of criticality within our data using this classical metric can be problematic: To grasp this kind of behavior, one needs to deal with very large systems arbitrarily close to the critical point. The number of networks available and their intrinsic small sizes makes this approach impracticable. However, there are other traces of criticality in percolation phenomena that revealed more robust when looking at real systems, such as the cluster size distribution (Bollobas, 1998; Brière et al., 2007; Koeze and Tighe, 2018; Newman et al., 2001). For example, in standard percolation in random graphs, cluster size distribution near the critical point displays a clearly defined power-law pattern $p_{c}\left(s\right)∼s^{-\gamma }$ with exponent γ=3/2. As a consequence, the average cluster size – excluding the giant cluster in the computations – diverges at the critical point (Newman et al., 2001). For the specific study of rigidity transitions, these markers of criticality were also explored, but in slightly different network processes (Brière et al., 2007; Koeze and Tighe, 2018). We took this approach in order to elucidate whether the experimental data had traces of criticality. Numerical explorations with square lattices with side size L=35, containing N=1208 nodes show that, close to the critical point (found around $\left\langle C_{c}\right\rangle \approx 3.92$, $\left\langle k_{c}\right\rangle \approx 0.68$ for lattices of this size), the probability distribution of rigid cluster sizes other than the $GC^{G}$, $p_{c}\left(s\right)\equiv p\left(S_{k}=s|\left\langle k\right\rangle =\left\langle k_{c}\right\rangle \right)$, follows a well defined power law:

$$p_{c}\left(s\right)\propto s^{-\gamma },$$

with γ∼2.5, fitted with the method of maximum likelihood estimation, as described in (Hanel et al., 2017) (see Figure S3K). Furthermore, computing the rigid cluster size distribution for smaller graphs (of the same average size as experimental graphs), predicted similar exponents although with a finite-size correction, both features providing excellent fit to the data (Figures 3G and S3N).

This critical exponent implies, as opposed to standard percolation in random graphs, that the average cluster size converges in a value independent of the network size and connectivity around ⟨s⟩∼4 for the interval of connectivities under study, whereas at the critical point, the variance of the cluster size, $\sigma_{c}^{2}=\left\langle s_{c}^{2}\right\rangle -{\left\langle s_{c}\right\rangle }^{2}$, is expected to diverge with the size of the lattice (see Figure S3L). In particular, given the value of the exponent, one expects $\sigma_{c}^{2}\left(s\right)$ to grow as a function of the system size, N, as:

(1) $$\sigma_{c}^{2}∼\frac{1}{3-\gamma }\sqrt{N}-{\left\langle s_{c}\right\rangle }^{2}∼2\sqrt{N}-{\left\langle s_{c}\right\rangle }^{2},$$

where $\left\langle s_{c}\right\rangle ∼4$, which agrees with our computational findings (Figure S3M). Importantly, turning to experimental cell-contact network revealed a behavior fully consistent with these results, with both the position and amplitude of the peak of $\sigma_{c}^{2}\left(s\right)$ well-fitted by the rigidity percolation model in the absence of adjustable parameters.

It should be noted that previous works had also computed cluster size exponents, for slightly different phenomena, and found slightly different values of γ∼2.1 for non-deformable adhesive spheres (Koeze and Tighe, 2018; Lois et al., 2008) and subcritical (γ∼2.2) or supercritical (γ∼2.7) regimes of self-organized, less stochastic network growth (Brière et al., 2007).

#### Rigidity phase transition and response of the system against deformations

An important insight of the rigidity percolation is its prediction on the rheological properties of the tissue. In particular, at the rigidity percolation critical point, networks acquire a non-zero elastic modulus upon deformation (matching the shear response of sphere packings) (van Hecke, 2010). Below the critical point of rigidity, small deformations of the network may be performed at no energy cost. On the contrary, beyond the critical point of rigidity, the network as a whole, starts exerting non-negligible force against any deformation. Similarly, work on the influence of weak attractive forces on the rheology of colloidal suspension predict a divergence of viscosity at a critical particle fraction (Krishnamurthy and Wagner, 2005), after which elasticity should be observed. In our experimental system, however, the tissue behaves as a viscous fluid even for very high connectivity values (Figure 1F, or alternatively very high cellular fractions, Figure S1E). In fact, beyond the rigidity percolation critical point, viscosity varies relatively little with further increase of connectivity (Figure 1F), so that even fully connected graphs display viscous rheology under micropipette aspiration. Given that such transition from elasticity to viscosity is frequent for biological material undergoing turnover (Ranft et al., 2010) or undergoing passive or active topological re-arrangements (T1 transitions), and that re-arrangements and finite contact times were observed in our system (Petridou et al., 2019), we explored the idea that incorporating in a toy model turnover would result in viscosity behaving similarly as elasticity (i.e., below the percolation transition, neither elastic or viscous network can resist deformation while above both can). We start by recapitulating the response of a purely elastic network as previously described, using a large triangular lattice with the bottom layer fixed in space with links as springs of elastic constant K=1. Given that $l_{0}$ is the rest length of the spring, force balance on a given (overdamped) node $v_{i}$ at position ${\vec{n}}_{i}$ leads to the following differential equation:

$$\underset{j}{\sum }\left(l_{ij}-l_{0}\right){\vec{r}}_{ij}+\rho \frac{d{\vec{n}}_{i}}{dt}=0,$$

where ρ is the damping parameter of the spring, j is an index summing over all neighbors of i, $l_{ij}$ the length of the link connecting nodes $v_{i}$ and $v_{j}$ and ${\vec{r}}_{ij}$ the unit vector associated to this link. The N nodes therefore define a system of N coupled differential equations determining the evolution of the network under potential external forces or deformations. To infer the linear response of the lattice against deformations, we complement this with two boundary conditions: i) we quasi-statically apply a small lateral displacement of the top layer by δx (see Figure 1H) small enough to grasp the linear response against this deformation performed by the network and ii) we fix the position of the bottom layer, to calculate the force $F_{0}$ exerted by this layer, against the imposed displacement at the top. This is similar to rheological assays with controlled strain.

In rigidity percolation, elastic networks cannot transmit forces below the critical point. Numerical experiments testing $F_{0}$ against ⟨k⟩ confirmed this, showing that $F_{0}$ displays negligible values until $\left\langle k_{c}\right\rangle$ (Figure S1J). For connectivity values above the rigidity percolation threshold $\left\langle k_{c}\right\rangle$, the transmitted force $F_{0}$ (i.e., elastic modulus) increases linearly with ⟨k⟩, again as expected from rigidity percolation in networks, as well as sphere packing under shear (van Hecke, 2010).

We now include the dynamic nature of cell-cell contacts to a permanent energy dissipation that will provide the network a viscous behavior. In general, viscosity η can be defined as:

(2) $$\eta ∼\frac{F_{0}}{v},$$

where $F_{0}$ is the force exerted over the system to deform it and v the velocity of the part of the network where this force is applied, as a response to it. In networks with turnover, energy is released spontaneously, enabling the network to be deformed irreversibly. The simplest way to implement such energy dissipation is to consider breaking and healing of bonds at a constant rate 1/τ in our system, which will give rise to viscous networks as measured experimentally. In this framework, a bond with rest length $l_{0}$ with actual length l may break and heal again with timescale τ, resetting the rest length to $l\prime_{0}=l$. In the case the network is subject to some external force/deformation, these dynamics will result in an irreversible deformation. In the case the network is subject to a constant force $F_{0}$ e.g., pushing up the top layer, the network will reach a regime where it will flow at constant speed υ depending on τ. Let us analyze this situation and how it projects into viscosity. Now assume that energy is dissipated due to a relaxation of the rest length of the springs occurring stochastically with an average time window of τ. In this case, there is a constant, C≡Δt/τ=1/τ, that determines the relation between velocity and τ, leading to Equation 2 to read $\eta ∼\tau \frac{F_{0}}{C\Delta y}.$ Since $F_{0}=K\Delta y$, one can rewrite the above expression as $\eta ∼\tau \frac{K}{C}$, which relates the relaxation time and the viscosity, providing therefore a very simple framework to explain viscous behaviors observed in our experiments. A crucial point is that, at the linear order, we expect the response to be similar to elastic networks with respect to the critical point, which we proceeded to check. To that end, we followed the following modeling criteria:

• We consider random triangular lattices G(V,E) with increasing connectivity and we deformed them by displacing all the nodes of the upper layer a distance δx (imposed shear), keeping the y coordinate constant for this layer.
• The total number of integration time steps $T_{e}$ was chosen such that a fully connected, elastic lattice of the same size than the ones used by the simulation reached a stationary state in terms of force propagation through the whole network under the displacement of δx of the nodes belonging to the upper layer.

We performed numerical tests under the above considered conditions. We generated random triangular lattices of size N∼248, L=16, $l_{0}=1$, K=1, and we performed a permanent displacement of the top layer of δx=0.01. ⟨k⟩ ranged from 0.2 to 1 in 20 equidistant connectivity intervals, and we performed 200 replicas per connectivity value. Assuming that the equilibration time of the elastic network is $T_{e}$, we considered three cases: i) elastic network ii) A viscous network whose links have an average lifetime of $\tau ∼2T_{e}$ and iii) a viscous network whose links have an average lifetime of $\tau ∼T_{e}$ (Figures 1H and S1J). Calibration was performed under the criteria that force distribution should reach a stationary pattern in the fully connected elastic network of this size. Under that criteria, we found that $T_{e}=600\delta t$ was enough.

#### Modeling of the biophysics of cell-cell contacts in rigidity transitions

To quantify the adhesive properties of the cells, we compute the ratio between cell-cell and cell-fluid surface tensions ($\gamma_{cc}$ and $\gamma_{cf}$, respectively) following the methodology developed in (Maître et al., 2015; Turlier et al., 2014). Let us suppose two cells, whose geometry in isolation is spherical with identical radius $R_{c}$. When we put them in contact, the balance of surface tensions will create a circular region of contact, and the contact point will define an angle θ between the two cell membranes and the fluid (Figure 4A) of the main text for a schematic. Following Maître et al., 2015, the area of contact between cells is:

$$A_{cc}=\pi {\left[R_{c}sin\left(\frac{\theta_{e}}{2}\right)\right]}^{2}.$$

In turn, the area of the cells in contact with the fluid is related to the contact angle as:

$$A_{cf}=2\pi R_{c}^{2}\left[1+cos\left(\frac{\theta_{e}}{2}\right)\right].$$

According to these two equations, the energy of these two cells in contact is given by:

$$E=2\gamma_{cf}A_{cf}+\gamma_{cc}A_{cc}.$$

From the above relation, it can be shown (Maître et al., 2015) that the relation between surface tensions when the system reached an energy minimum is given by:

$$\frac{\gamma_{cc}}{2\gamma_{cf}}=cos\left(\frac{\theta_{e}}{2}\right),$$

Therefore, by defining the relative cell-cell tension α (which decreases for increasing cell adhesion) as:

$$\alpha \equiv \frac{\gamma_{cc}}{2\gamma_{cf}},$$

one has a single parameter depicting the relation between surface tensions, which can be extracted from experimental data by computing the angle of contact between cells at the fluid interface. This analysis shows that relative cell-cell tension α (resp. cell-cell adhesion) decreases (resp. increases) at the fluidization point (Figure 4B and B’), and displayed a strong anti-correlation with tissue viscosity across time and conditions (Figure 4D).

To relate these changes to the changes of cell connectivity observed from blastoderm network reconstruction, we generalized this doublet model to configurations of arbitrary cell numbers. In particular, we used a two-dimensional toy model of 4 cells with equal volumes and tension, organized spatially into a rhombus with 4 links. This mirrored one of the simplest floppy topologies sketched in Figure 4A. We started from non-adhesive spheres (α=1), and gradually decreased cell-cell tension (increased adhesion). This caused an expansion of cell-cell doublet contacts, which above a critical threshold in relative cell-cell tension α merged to form tri-cellular vertices, triggering the spontaneous creation of a cell contact that makes the system to transit from floppy to rigid, in topological terms (Figure 4A). The trigger of rigidity is found at around α≈0.85–0.9, although this value depends on the exact initial configuration used, and displays hysteresis upon increasing again α above the threshold. Finally, we also considered disordered arrangement of cells, and found that the threshold value toward rigidity was located around the same ranges of α (Figure S4E). Simulations to compute equilibrium arrangements of cells have been performed using the free available software ‘Surface Evolver’ (Brakke, 1992).

### Quantification and statistical analysis

#### Processing and quantifications of experimental data

All acquisition data were processed using Fiji (NIH) and/or Imaris 9.0.

*Cell connectivity (< C > )* was calculated in each confocal section as total number of contacts (defined as described in connectivity map reconstruction) divided by the total number of cells in the image. *Normalized connectivity (< k > )* was calculated in each confocal section as connectivity C divided by the maximum potential connectivity (k_max_) (computed as described in Supplementary Information Text). *3D number of contacts per cell* was calculated in Imaris by randomly choosing blastoderm cells, followed by manual identification of the cell-cell contacts using the “Ortho-slice” and “measurement” tools (Figures S1L and S1L’).

The *mean squared relative displacement (MSRD)* was calculated from individual 3D cell trajectories following the nuclei position in Imaris of two cells (Cell_A_, Cell_B_) that were in contact at the first time point. The relative xyz position between the Cell_A_ and Cell_B_ was defined as:

$$p_{rel}=\surd \left({x_{rel}}^{2}+{y_{rel}}^{2}+{z_{rel}}^{2}\right)$$

where $x_{rel}=x_{A}-x_{B}$, $y_{rel}=y_{A}-y_{B}$, $z_{rel}=z_{A}+z_{B}$. The MSRD was defined as:

$$MSRD={\left(p_{relt1}-p_{relt0}\right)}^{2},$$

with *t*_*0*_ being the first time point and *t*_*1*_ 3 min later (Figures S3I and S3I’). MSRD for the different time periods was defined as t_1_ = −30 and t_0_ = −60 for t-60 min, t_1_ = 0 and t_0_ = −30 for t-30 min, t_1_ = 30 and t_0_ = 0 for t0 min, t_1_ = 60 and t_0_ = 30 for t30 min and t_1_ = 90 and t_0_ = 60 for t 60min (Figures S1B and S1B’).

*Cell fraction (cf.)* was calculated as:

cf=1−ff,whereffisfluidfraction.

*ff* was calculated from 2D confocal sections at the 1^st^-2^nd^ deep cell layers, where the interstitial fluid channel (labeled with Alex Fluor 647 Dextran) was first converted into a binary image and then processed by a median filter of 2-pixel radius in Fiji. A signal intensity histogram analysis of the whole binary image was performed in Fiji (range of 256 different signal intensities, with 256 being the maximum), and the *ff* was obtained by the average gray value of the histogram normalized to the max signal intensity of the histogram (256) (Figure S1E).

*Cell shape index (q)* analysis was calculated from 2D confocal sections at the 1^st^-2^nd^ deep cell layers, where the cell perimeter *P* and cell area *A* were obtained using the freehand selection tool to draw the cell perimeter and q was defined as previously described (Bi et al., 2016) with $q=\left(\frac{P}{\sqrt{A}}\right)$ (Figure S1C). *Cell area* analysis shown in Figure S5C was also performed using the freehand selection tool to draw the cell perimeter in Fiji. *Cell area variability* shown in Figure S5D was expressed as the coefficient of variation CV=sd/mean of the cell area measurements shown in Figure S5C.

*E-cadherin expression* was quantified by the fluorescent intensity of E-cadherin levels as judged by antibody staining. Control-morphant and *e-cadherin*-morphant embryos were processed for immunofluorescence within the same tube. The average fluorescence intensity was calculated in Fiji within an ROI covering the central deep-cell blastoderm (Figure S3C).

The cell-cell contact angle, θ_*e*_, was calculated in degrees from 2D confocal sections using the angle tool in Fiji, as indicated in Figure 4B, and then converted to radians. The cell-cell tension α was then calculated as:

$$\alpha =cos\left(\theta_{e}/2\right).$$

The spatial *heterogeneity in cell connectivity (k*_*het*_*) and fraction of dividing cells (fd*_*het*_*)* (Figures 5A and 5A’) was analyzed by computing the variance of the two parameters between the quadrants of the 2D confocal sections used for the tissue rigidity analysis. For *k*_*het*_, in each quadrant (Area I, II, III, IV), the normalized connectivity was calculated as described above (k_I_, k_II_, k_III_, k_IV_) and then the variance was calculated between k_I_, k_II_, k_III_, k_IV_. For *fd*_*het*_, in each quadrant the fraction of dividing cells was calculated as the number of mitotic nuclei (excluding cytokinesis stage) divided by the total number of nuclei in the plane (fd_I_, fd_II_, fd_III_, fd_IV_). In order to compare the variance in *fd* between WT and Chk1 overexpressing (oe) embryos displaying different number of cells, the standard deviation between the quadrants was first normalized using a binomial test. The probability *q* of a cell dividing was approached as q=n/N, with *n*, total number of mitotic nuclei and *N*, total number of nuclei. This defines the null model underlying the statistical test. The probability that a dividing cell is in a given quadrant is 1/4. The probability that *N*_*i*_ cells in quadrant *i* from which *n*_*i*_ are dividing, p(N_i_, n_i_) is then given by:

$$p\left(N_{i},n_{i}\right)=p\left(N_{i}\right)p\left(n_{i}|N_{i}\right).$$

Here, $p\left(N_{i}\right)$ is a binomial distribution with parameter 1/4 and number of trials N. $p\left(n_{i}|N_{i}\right)$ is a binomial distribution with parameter q and number of trials *N*_*i*_. The expected average number of dividing cells per quadrant is then $<n>=\frac{qN}{4}=n/4$. The expected standard deviation of dividing cells per quadrant is:

$$std\left(nullmodel\right)=\left(\frac{1}{2}\right)\sqrt{Nq\left(1-q\right)}=\left(\frac{1}{2}\right)\sqrt{n\left(1-q\right)}.$$

The absolute value of the z-score for each quadrant and the number of dividing cells is:

$Z_{i}=\left(K_{i}-<n>\right)/std\left(null\right)$, with *K*_*i*_, the observed number of dividing cells in quadrant *Q*_*i*_. The spatial heterogeneity coefficient, SHC, is then $SHC=var\left(Z_{I},Z_{II},Z_{III},Z_{IV}\right)$, plotted in Figure 4B.

The *fraction of dividing cells* shown in Figure S5A’ was calculated as the number of mitotic nuclei (except cytokinesis phase), divided by the total number of nuclei in the 2D confocal sections. The *relationship between connectivity and fraction of dividing cells* was analyzed during the meta-synchronous cell cleavage cycles (−60 to 0 min for WT embryos, −60 to 60 min for Chk1 oe embryos) in higher magnification 2D confocal sections by following every 5 min a randomly selected cell and its direct neighbors (presence of a cell-cell contact). The increase in the fraction of dividing cells and the reduction in the relative number of contacts every 10 min was plotted in Figure S5B.

The quantification of differences in *the viscosity fold-change* between WT and Chk1 oe embryos over time was calculated from viscosity measurement experiments where all embryos originate from the same batch. The relative change in the viscosity value was calculated as $\eta_{rel}=\eta_{t1}/\eta_{t2}$, with η being the viscosity values and the time interval between t1 and t2 being < 10 min. From each batch the percentage of embryos exhibiting $\eta_{rel}$ 0-1, 1-5, 5-10 and > 10 was calculated for each time point (Figure S4I).

The *variability in the viscosity* was expressed as the coefficient of variation CV=sd/mean for each time point of viscosity measurements for the same number of embryos between WT and Chk1 oe embryos (Figure S5J).

The *robustness viscosity factor* was expressed as the inverse of the coefficient of variation (Figure 5G).

Cell-cell *contact length fluctuations* were calculated from 2D confocal time series acquired using the Fast Airyscan mode (10 s interval, 10 min time lapse duration). For each time point, the contact length was measured using the free-hand line tool in Fiji (Figure S5K). Then, contact lengths were normalized to the average of the contact lengths during the 10 min duration and the frequency distribution (in percentage) was calculated in GraphPad prism (contact lengths were binned in 0.1 intervals) (Figure S5K’). The contact length fluctuations in Figure S5K’’ was expressed as the coefficient of variation, CV=sd/mean, for each contact during the 10 min measurement, and the average of CV of 10 contacts per time point and condition was plotted. The kymographs shown in Figure S5K were generated in Fiji, using the KymoResliceWide plugin.

#### Statistical analysis of experimental data

The statistical analyses were performed with GraphPad Prism 6.0. Statistical details of experiments are reported in the figures and figure legends. Sample size is reported in the figure legends, and no statistical test was used to determine sample size. The biological replicate is defined as the number of embryos or independent batches of embryos, as stated in the figure legends. No inclusion/exclusion or randomization criteria were used and all analyzed samples are included. Unless differently stated in the figure legends, the graphs show mean ± sem and the error bars are calculated and shown based on the number of cells or embryos, as indicated. The statistical test used to assess significance is stated in the figure legends and was chosen after testing each group with the normality distribution test D’Agostino. For comparing two groups, a two- tailed Student’s t test for parametric distributions and a Mann–Whitney U-test for non-parametric distributions were used. To compare more than two groups, either an analysis of variance (ANOVA) or Kruskal–Wallis test for parametric and non-parametric distributions, respectively, was used. No blind allocations were used during the experiments or in the analysis. At least more than three independent experiments were performed except for the data shown in Figure S3C (embryos from two different embryo batches, in which an independent experiment was defined as the embryo batch). This information is also stated in the figure legends.

#### Construction of the universal curves for the GC size

To obtain the universal curve accounting for the expected size of the GC in random triangular lattices along connectivity values, we first computed the average size of all experimental networks (except the Chk1 overexpression networks, which we treat separately as discussed below), leading to ⟨N⟩∼100. We then generated model lattices for 50 equidistant connectivity values from 0.2 to 1 of size ∼⟨N⟩. For every connectivity value, we performed 50 replicas and computed the average and standard deviation of the GC.

To compute the model curve appearing in Figure 1G’, we proceeded exactly as above with different lattice sizes. In particular, we considered the following network sizes N=46,N=116,N=315,N=613 respectively.

#### Average and variance of cluster sizes other than the GC

To obtain the curve accounting for the expected sizes of the clusters other than the GC in real networks along connectivity values, we considered 50 equidistant connectivity values from 0.2 to 1. We then created, for each connectivity point, 50 lattices with the same criteria as above, computed the overall cluster size average and variance and compared them to the curve obtained for real networks.

#### Cluster sizes at criticality

To produce the part corresponding to real data of the plot shown in Figures 3F and 3G, we first considered that, for networks of size ∼100, the peak in the $\sigma_{c}^{2}\left(s\right)$ – which we take as the indicator of the critical point – was located around $\left\langle k_{c}\right\rangle ∼0.72$. The cluster size analysis near criticality was performed by collecting the cluster sizes other than the GC for all the networks whose ⟨k⟩ laid in the interval $\left\langle k_{c}\right\rangle \pm 0.015$ and then computing the statistics. This interval ensured certain degree of resolution as well as certain volume of data to perform statistics. According to that criteria, we found that 30 of the studied experimental networks were in the critical regime. We then computed the same metrics with the same criteria in computational networks of the same average size.

#### Modeling global WT cluster size distribution

Cluster size distribution offers a detailed map of the potential heterogeneities and deviations from pure randomness in the connectivity patterns. To avoid confusion, it is worth to emphasize that here we are considering the whole range of connectivity values, not only those near the critical point. As we see in the results, WT cluster size distribution can be fairly approximated using random lattices as models. However, for Chk1 overexpressing (oe) embryos the case is different: The cluster size distribution departs clearly from the expected behavior if the underlying null model are random networks. We needed to use, instead, a model lattice that creates spatial correlations, as the one defined above. Below we detail the numerical experiments performed for the cluster size analysis.

To perform reliable statistics in order to derive the cluster size distribution, we needed to be more detailed than in the above computations, given the small nature of the system and the fine-grained observable we are dealing with. To that end, we refined the generation of the null model for lattice structure.

We created $S_{WT}=92$ uncorrelated lattices, which is the exact number of WT real networks in the sample. Lattice sizes were picked at random following the lattice size distribution $p_{WT}\left(N\right)$ obtained from real data. The average connectivity of each generated random lattice was chosen at random uniformly within the interval ${\left\langle k\right\rangle }_{min,WT}=2.5$ and ${\left\langle k\right\rangle }_{max,WT}=4.45$.

With the above methodology, we create a null model for the topology of (random) lattices belonging to the WT set. We performed 30 replicas of the whole process and plotted the results in Figures 5C and S5E.

#### Modeling global Chk1 oe cluster size distribution

For the modeling of cluster size distribution of the Chk1 oe data (Figure 5C) of the main text, we used the method for generating correlated lattices with data parameters L,⟨k⟩, defined as above, and λ as the single free parameter. We created $S_{Chk1}=95$, which is the exact number of Chk1 oe real networks in the sample. Lattices sizes were picked at random following the lattice size distribution $p_{Chk1}\left(N\right)$ obtained from real data. The average connectivity of each generated random lattice was chosen at random uniformly within the interval ${\left\langle k\right\rangle }_{min,Chk1}=2.87$ and ${\left\langle k\right\rangle }_{max,Chk1}=4.58$. With the above methodology, we create a null model for the topology of (correlated) lattices belonging to the WT set. We performed 30 replicas of the whole process and plotted the results in Figure S5E.

To find the optimal fit of the parameter λ between real data and simulated results we used the method of minimization of the Jensen-Shannon divergence (Cover and Thomas, 2006) between real and numerical data. The global optimum was found in λ=0.47, implying a spatial correlation in the distribution of cluster sizes of a few cell length, which is quite large given the finite size of the experimental networks, providing an explanation for the much less uniform viscosities measured experimentally in Chk1 oe embryos during the studied period.

#### Analysis of the second cluster size

This analysis was performed identically for the WT and Chk1 oe datasets. To check the statistical relevance of the potential deviations of the size of the second largest cluster, for each real network, we generated 100 replica networks with approximately the same size and average connectivity and, from this population, we computed the expected value for the second largest cluster. We then aggregated the real networks in their respective different time points and computed the average size and standard deviation of the second cluster. We did the same operation using the expected second cluster sizes we computed before. Consistent with the deviation from the expected cluster size distribution observed in Chk1 oe embryos, we observe that, in general, the size of the second cluster is bigger than the expected cluster size (Figures S5G and S5G’).

## Supplemental information

Table S1. Rigidity analysis of all experimental networks, related to Figures 2, 3, and 5
